# Enhancing the Behaviour Change Wheel with synthesis, stakeholder involvement and decision-making: a case example using the ‘Enhancing the Quality of Psychological Interventions Delivered by Telephone’ (EQUITy) research programme

**DOI:** 10.1186/s13012-021-01122-2

**Published:** 2021-05-14

**Authors:** Cintia L. Faija, Judith Gellatly, Michael Barkham, Karina Lovell, Kelly Rushton, Charlotte Welsh, Helen Brooks, Kerry Ardern, Penny Bee, Christopher J. Armitage

**Affiliations:** 1 Division of Nursing, Midwifery and Social Work, School of Health Sciences, Faculty of Biology, Medicine and Health, University of Manchester, Manchester Academic Health Science Centre, Manchester, UK; 2 Clinical Psychology Unit, Department of Psychology, University of Sheffield, Sheffield, UK; 3 Department of Health Services Research, Institute of Population Health Sciences, University of Liverpool, Liverpool, UK; 4 Department of Psychology, University of Sheffield, Sheffield, UK; 5 Manchester Centre for Health Psychology, Division of Psychology and Mental Health, School of Health Sciences, Faculty of Biology, Medicine and Health, University of Manchester, Manchester, UK; 6 Manchester University NHS Foundation Trust, Manchester Academic Health Science Centre, Manchester, UK; 7 NIHR Greater Manchester Patient Safety Translational Research Centre, Manchester, UK

**Keywords:** Behaviour Change Wheel, Mental health services, Improving Access to Psychological Therapies (IAPT), Psychological interventions, Guided-self-help, Telephone, Intervention development, Implementation, Remote working

## Abstract

**Background:**

Using frameworks such as the Behaviour Change Wheel to develop behaviour change interventions can be challenging because judgement is needed at various points in the process and it is not always clear how uncertainties can be resolved. We propose a transparent and systematic three-phase process to transition from a research evidence base to a behaviour change intervention. The three phases entail evidence synthesis, stakeholder involvement and decision-making. We present the systematic development of an intervention to enhance the quality of psychological treatment delivered by telephone, as a worked example of this process.

**Method:**

In phase 1 (evidence synthesis), we propose that the capabilities (C), opportunities (O) and motivations (M) model of behaviour change (COM-B) can be used to support the synthesis of a varied corpus of empirical evidence and to identify domains to be included in a proposed behaviour change intervention. In phase 2 (stakeholder involvement), we propose that formal consensus procedures (e.g. the RAND Health/University of California-Los Angeles Appropriateness Methodology) can be used to facilitate discussions of proposed domains with stakeholder groups. In phase 3 (decision-making), we propose that behavioural scientists identify (with public/patient input) intervention functions and behaviour change techniques using the acceptability, practicability, effectiveness/cost-effectiveness, affordability, safety/side-effects and equity (APEASE) criteria.

**Results:**

The COM-B model was a useful tool that allowed a multidisciplinary research team, many of whom had no prior knowledge of behavioural science, to synthesise effectively a varied corpus of evidence (phase 1: evidence synthesis). The RAND Health/University of California-Los Angeles Appropriateness Methodology provided a transparent means of involving stakeholders (patients, practitioners and key informants in the present example), a structured way in which they could identify which of 93 domains identified in phase 1 were essential for inclusion in the intervention (phase 2: stakeholder involvement). Phase 3 (decision-making) was able to draw on existing Behaviour Change Wheel resources to revisit phases 1 and 2 and facilitate agreement among behavioural scientists on the final intervention modules. Behaviour changes were required at service, practitioner, patient and community levels.

**Conclusion:**

Frameworks offer a foundation for intervention development but require additional elucidation at each stage of the process. The decisions adopted in this study are designed to provide an example on how to resolve challenges while designing a behaviour change intervention. We propose a three-phase process, which represents a transparent and systematic framework for developing behaviour change interventions in any setting.

**Supplementary Information:**

The online version contains supplementary material available at 10.1186/s13012-021-01122-2.

Contributions to the literature

- Using systematic guidelines to develop behaviour change interventions requires judgements, which can be opaque.
- We propose a three-phase process, namely, evidence synthesis, stakeholder involvement and decision-making to move from the evidence base to the intervention involving and incorporating stakeholder perspectives into the design process.
- We believe this approach represents a transparent and systematic framework for developing behaviour change interventions in any setting.

## Introduction

The Behaviour Change Wheel [1] is a systematic method that is endorsed as a key theoretical framework by the National Institute for Health and Care Excellence (NICE) [2] for the development and evaluation of behaviour change interventions. The Behaviour Change Wheel [1] makes explicit many of the processes involved in the development of behaviour change interventions, the starting point for which is understanding the behaviour (e.g. identifying what needs to change), followed by identifying intervention options and identifying content. The Behaviour Change Wheel [1] also provides tools for negotiating each of these phases of intervention development. For example, the capabilities (C, physical and psychological), opportunities (O, physical and social) and motivations (M, automatic and reflective) model of behaviour (B: COM-B) captures the key drivers of behaviour that may need to change, and APEASE articulates the criteria (acceptability, practicability, effectiveness/cost-effectiveness, affordability, safety/side-effects and equity) by which decisions about intervention content (e.g. mode of delivery) should be made [1].

However, the operative word in relation to the Behaviour Change Wheel [1] is *method*, and although the Behaviour Change Wheel suggests a series of broad steps to follow in developing interventions it is not a ‘magic bullet’ ([1]: p.27). This means that every step in the process of intervention development is not prescribed and that developers need to ‘be comfortable using judgement’ ([1]: p.125) to decide what suits best for the context. There is currently a lack of worked examples of how to identify and then negotiate the likely numerous points at which judgement is needed to develop coherent and effective interventions. This is important, because there are numerous examples of the Behaviour Change Wheel [1] being used to develop interventions that lack of transparency in how key decisions were made. For example, although Barker et al. [3] rigorously apply the Behaviour Change Wheel [1], it is not clear how they synthesised their varied corpus of evidence to arrive at their final intervention (‘we then combined…’, p. 491). The lack of specified opportunities for stakeholder involvement, a core philosophy of contemporary health care [4], has been acknowledged as a specific weakness in the implementation of the Behaviour Change Wheel approach [5].

## Aims

**Fig. 1:**
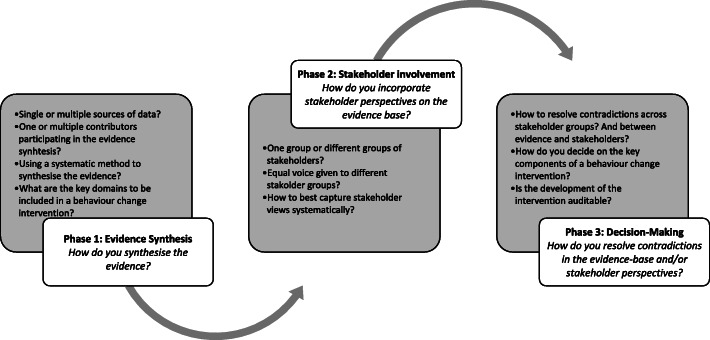
The process for developing a behaviour change intervention: from the evidence to the intervention

The aim of this paper is to propose a transparent and systematic process for intervention development: (1) evidence synthesis, how to bring together evidence from multiple sources; (2) stakeholder involvement, how to incorporate stakeholder perspectives on the evidence base; and (3) decision making, how to resolve contradictions in the evidence base and/or stakeholder perspectives (see Fig. 1). We describe how the transition from empirical evidence (evidence synthesis) to the development of behaviour change intervention materials (decision-making) can be negotiated, considering key stakeholder priorities (stakeholder involvement).

Our worked-example reports on each phase of the development of a behaviour change intervention designed to enhance the quality of psychological interventions delivered by telephone (EQUITy research programme). Mental health problems are affecting an increasing number of people [6] and a critical step in maximising the availability, accessibility and cost-effectiveness of evidence-based mental healthcare is to develop and implement high quality, safe alternatives to face-to-face treatment. Although meta-analysis, randomised trials and large observational studies have shown telephone-delivered psychological interventions to be clinically and cost-effective compared to face-to-face delivery [7–9], their routine uptake in practice and their sustained use by patients has lagged behind this evidence [10, 11]. Qualitative enquiry has shown the root of this delay to be a multifaceted problem, driven not just by the behaviour of patients themselves, but also by the practitioners delivering psychological services, and the people who manage them [12–15]. Enhancing the quality of psychological interventions delivered by telephone thus provides a rich and contemporary relevant context to illuminate some of the processes involved in behaviour change intervention development.

### Phase 1: Evidence synthesis

#### Introduction

Evidence is at the heart of the Behaviour Change Wheel [1] approach to intervention development, but questions inevitably arise as to what types of evidence are relevant, how much of each type of evidence is necessary and how might intervention developers best prioritise or bring different types of evidence together. We have labelled the first phase of the development of our worked-example intervention ‘evidence synthesis’. Evidence synthesis may be required at each phase of intervention development specified in the Behaviour Change Wheel [1]: it can be used to identify what needs to change as well as what modes of delivery might be most effective.

Systematic reviews and meta-analysis are at the top of the evidence hierarchy [16, 17] and are extremely useful when they are available, but they rarely cover every aspect of the intervention that needs to be developed (e.g. from selecting target behaviours to identifying mode of delivery). Thus, when embarking on intervention development, there are often gaps in existing knowledge that need addressing rapidly. This might mean additional data collection (qualitative and/or quantitative) and/or rapid evidence reviews are required to fill gaps in systematic reviews. However, the challenge is to identify a structured way in which to synthesise such evidence from multiple sources that maximises input from people who may not have a behavioural sciences background, let alone familiarity with the Behaviour Change Wheel [1].

#### Methods

##### How to bring together evidence from multiple studies/sources?

Ideally, and upholding the central ethos of evidence-based health care, synthesis would be achieved by conducting a mixed-methods meta-synthesis based on published papers. In addition, training stakeholders in research methods and the Behaviour Change Wheel so that they could fully and robustly participate in evidence synthesis would integrate and enable coproduction. Often however, intervention needs to be articulated through a lens of tacit demand or more acute system changes, evidence gaps, publication delays or funding windows that introduce time pressures into real-world scenarios. In such situations, judgment is needed [18, 19].

Table 1 summarises the steps needed to negotiate the evidence synthesis phase, alongside suggestions as to how to strike a balance between the ideal of mixed-methods meta-synthesis with the realities of intervention development. Key features of the process include the idea that the personnel involved should be as broad as possible and the recommendation that COM-B be used to help structure people’s responses. We have found that COM-B is easily communicated to non-experts. Additional Files 1, 2, 3 and 4 provide a worked example of the approach in relation to improving quality and engagement on telephone working and increase likelihood of effective and successful implementation of an intervention in clinical practice.

**Table 1:** Evidence synthesis phase

	Ideal	Possible compromises	Worked example
Evidence collection	Mixed-methods meta-synthesis based on published papers	• Rapid primary data collection • Rapid reviews	• 5 primary qualitative studies • 1 rapid review • 1 literature mapping exercise
Personnel	Widest possible grouping of fully trained stakeholders design and conduct the mixed-methods meta-synthesis	• Multidisciplinary research team • Behavioural scientists plus practitioners	• Face-to-face meeting of multidisciplinary research team (*N* = 16) including behavioural scientists, academics, psychologists, mental health practitioners and the patient and public involvement lead
Input	Recommendations based on mixed-methods meta-synthesis	Accessible summaries of the key findings and recommendations for intervention development from individual studies	Two weeks prior to face-to-face meeting, the full programme team (*N* = 27) received: • Two slides for a 5-min presentation • Single-page summaries of the key findings and recommendations for each of the 7 studies and comments invited.
Identify key domains to be included in intervention	Already present in mixed-method synthesis conclusions	Use COM-B to structure people’s identification of key domains, which can be completed by: • Individuals • Small group • Facilitated large group discussions	Face-to-face meeting attendees used post-it notes to categorise intervention domains into the six COM-B areas: • Individually • In small groups mixed by experience/expertise • In facilitated large group discussion
Cross-validation	Experts cross-check evidence synthesis phase with knowledge and experience	Experts cross-check evidence synthesis phase with knowledge and experience	Identification of potential barriers/enablers to behaviour change identified via a scoping search of previously published literature conducted by PB and KL

#### Results

##### What was the output from the evidence synthesis phase?

The evidence synthesis phase resulted in 93 domains of people’s capabilities, opportunities and motivations that needed to change to improve delivery of psychological interventions by telephone (Additional File 5). From the 93 domains, 40 were related to capabilities (all psychological), 31 to opportunities (18 physical and 13 social) and 22 to motivations (16 reflective and 6 automatic). The target domains involved changes at practitioner, patient, service and community levels.

In brief, changes at a *practitioner* level included mainly development/enhancement of telephone-specific skills (capabilities); changes in negative beliefs and emotions associated to telephone treatment and acknowledgement of professional role expectations (motivation). Changes at *patient* and *community* levels were related to increased knowledge/awareness of the different types of psychological interventions and its different modes of delivery (capabilities). Changes at a *service* level consisted of providing standardised and clear guidelines on procedures to deliver psychological treatment by telephone and clarify drivers for its use (capabilities); adjust working environment (e.g. reduce noise) (opportunities), ensure resources (e.g. good quality headsets) (opportunities), provide social support for working remotely (e.g. assessment and monitoring of telephone performance) (opportunities) and continued professional development (opportunities). The COM-B domains correspond closely with those that are articulated in the Theoretical Domains Framework (TDF) [20], and evidence analysed using different theoretically informed approaches was synthesised effectively and the relevant behaviour change domains identified.

Results of the evidence synthesis meeting were then cross-validated with broader literature relevant to the target group, identified prior to progressing to phase 2. As part of the cross-validation, we generated a list of potential barriers/enablers to behaviour change identified via a scoping search of previously published literature conducted by members of the programme team (PB and KL) (see tables in Additional Files 1 and 2) and feedback gathered from clinical work. We included studies focused on the same target populations (patient, practitioners) and health intervention (telephone-delivered psychological interventions), irrespective of whether or not they used a TDF analysis. We then compared our generated list of extrapolated influences to the specific evidence we had generated to inform the design of the intervention. Team consensus considered all potential influences to be adequately represented by our evidence synthesis output, and no further additions to our data set were required.

### Phase 2: Stakeholder involvement

#### Introduction

Patient and public involvement (PPI) plays an active role in health and social care services and in research, and its importance is rising [4, 21]. The Behaviour Change Wheel approach [1] has been criticised for its lack of patient involvement [5]. Involvement of the public is needed to ensure development and improvement in service care is equal, efficient, effective, patient-centred, relevant and appropriate [22–24]. Further to the cross-validation exercise proposed at the end of phase 1, an additional sense-checking of these results with multiple stakeholder groups is necessary. The challenge is to ensure that different perspectives have equal contributions while developing a coherent intervention.

#### Methods

There are multiple ways in which stakeholders can be involved in the translation of evidence into practice, such as the Delphi method [25], the Nominal Group Technique [26, 27], and the Consensus Development Conference [28, 29], but most force participants to achieve consensus [30]. Forced consensus may inhibit creativity and lose important nuance in intervention design. We would therefore propose an adapted version of the RAND Health/University of California-Los Angeles Appropriateness Methodology (RAND/UCLA) [31], which does not force consensus and provides participants with opportunities to discuss and change ratings after further considerations. The RAND/UCLA method usually involves single groups of experts, but we would advocate adapting the methodology by: (a) considering all potential participants as experts, and (b) running RAND/UCLA exercises separately for different groups of stakeholders in order to capture the expertise of all stakeholders without inhibition (e.g. practitioners versus managers). Additional File 6 provides details on recruitment of stakeholders and describes RAND/UCLA procedures and adaptations.

Table 2 summarises the steps needed to negotiate the stakeholder involvement phase, alongside suggestions as to how to adapt the RAND/UCLA method to meet this purpose. Key features of the process include the idea that the personnel involved should not be restricted to ‘experts’, but that different stakeholder groups be treated separately. Additional File 6 provides a worked example of the approach in relation to improving quality and engagement on telephone working and increase likelihood of effective and successful implementation of an intervention in clinical practice. Additional File 7 includes demographic characteristics for the stakeholder groups. Experiences of stakeholders related to taking part in the meeting are included in Additional File 8. A questionnaire aimed to gather information at the stakeholder meetings on a proposed element of the behaviour change intervention (i.e. skills training) is included in Additional File 9.

**Table 2:** Stakeholder involvement phase

	Standard RAND/UCLA	Adapted RAND/UCLA	Worked example
Inputs	Series of systematic reviews/meta-analyses	Intervention domains from phase 1	93 domains identified in phase 1
Stakeholders	Experts	As broad as possible, being mindful of traditionally excluded groups	• Patients (*n* = 9) • Practitioners (*n* = 19) • Key informants (*n* = 15)
Number of rounds	3: • Individual ratings of appropriateness • Moderated group ratings of appropriateness • Individual ratings of necessity	3 per group of stakeholders: • Individual ratings of importance • Moderated group ratings of items rated ‘not important’ • Individual ratings of ‘how essential?’	Separate meetings of patients, practitioners and key informants completing: • Individual ratings of importance • Moderated group ratings of items rated 'not important' • Individual ratings of ‘how essential?’
Critical cut-offs	Median of = > 7 on 1–9 point Likert-type scales	Median of = > 7 on 1–9 point Likert-type scales	Median of = > 7 on 1–9 point Likert-type scales

#### Results

##### What was the outcome of the stakeholder involvement phase?

At the end of Round 1, ‘extremely important’ ratings (i.e. Median ≥ 7) had been given to 90.3% of domains by patients, 84.9% by practitioners and 90.3% by key informants. Following moderated discussion during Round 2, an additional 3.2% and 7.5% domains were rated ‘extremely important’ by patients and practitioners respectively; no additional domains were rated as extremely important by key informants. Details including ratings for Round 1 and Round 2 for each stakeholder group are included in Additional File 10, and minutes from moderated discussions are included in Additional File 11. Data from Round 3 showed that 89.2% of domains by patients, 89.2% by practitioners and 62.4% by key informants were rated as ‘essential’ (i.e. Median ≥ 7) and were taken forward to phase 3 (see Additional File 12a). Domains rated at Round 3 as ‘not essential’ (i.e. median < 7) are included in Additional File 12b. Experiences from each of the stakeholder groups about their participation in the meeting are included in Additional File 13.

### Phase 3: Decision-making

#### Introduction

The principal aims of the decision-making phase are to (a) address how to resolve any contradictions in the evidence base and/or across stakeholder perspectives, (b) choose which domains to include in the behaviour change intervention, (c) identify intervention functions, (d) propose behaviour change techniques [1] and (e) address demand while retaining applicability and practicality across diverse health settings/services. Consistent with the ethos of stakeholder involvement, ideally the decision-making phase would involve equitable contributions from all partners. However, the reality is that few intervention development programmes have the opportunity to train adequately all the stakeholders.

#### Methods

In the broader literature [32], typically final decisions on intervention content are made either by the behavioural scientist on the team or among a small number of members of the programme team. Sometimes the results are circulated and the decisions made for feedback to the wider team. With the luxury of time, key stakeholders could be trained and a larger-scale decision-making meeting could be held. Table 3 provides details on decision-making procedures, divided into what would ideally be done, possible compromises that could be made alongside some concrete instances from our worked example. Additional File 14 provides a more detailed worked example of the procedures taken in relation to improving quality and engagement on telephone working and increase likelihood of effective and successful implementation of an intervention in clinical practice.

**Table 3:** Decision-making phase

	Ideal	Possible compromises	Worked example
Personnel	Widest possible grouping of fully trained stakeholders	• Multidisciplinary research team • Behavioural scientists plus practitioners	Face-to-face meeting of an intervention development subgroup comprised of a behavioural scientist, principal investigators, programme manager, researcher, patient and public involvement lead
Input	Recommendations based on RAND/UCLA analyses of multiple stakeholder groups conducted separately	Recommendations based on RAND/UCLA analyses of single expert group	Three days prior to face-to-face meeting, intervention development subgroup (n=6) received output of phase 2
Identify intervention content	All stakeholder groups agree fully on intervention domains. Translate intervention domains into behaviour change techniques and modes of delivery using APEASE criteria	Decide on decision rules to aid choice of intervention domains. Translate intervention domains into behaviour change techniques and modes of delivery using APEASE criteria	Agree criterion of two or more stakeholder groups from phase two rating domains as ‘essential’ (i.e. Median ≥ 7 on round 3). Translate intervention domains into behaviour change techniques and modes of delivery using APEASE criteria
Final approvals	Full multidisciplinary research team and all stakeholders provided with intervention materials for feedback. Details of the behaviour change intervention described using the Template for Intervention Description and Replication (TIDieR)	Subgroup of multidisciplinary research team and stakeholders provided with intervention materials for feedback. Details of the behaviour change intervention described using the Template for Intervention Description and Replication (TIDieR)	Full multidisciplinary research team provided with intervention materials for feedback. Details of the behaviour change intervention described using the Template for Intervention Description and Replication (TIDieR)

#### Results

##### What was the outcome of the decision-making phase?

Fifty-five domains were rated as ‘essential’ across all the stakeholder groups and 25 across two of them (see Additional File 12a). Thus, 80 domains from the 93 initially proposed were included in the behaviour change intervention. Domains rated as ‘not essential’ (i.e. median < 7) by two or more stakeholder groups were not included in the intervention and are presented in Additional File 12b.

From the 80 domains agreed across the majority of stakeholder groups, 39 corresponded to capabilities (all psychological), 22 to opportunities (11 physical and 11 social) and 19 to motivation (16 reflective and 3 automatic). Changes in the behaviour of practitioners, patients, service leads and community members (e.g. general practitioners) were required. Using the APEASE criteria, it was decided that changes in practitioner behaviour would be addressed in training and that changes in other targets would be made through leaflets, posters and booklets. Changes in behaviours across the different target levels are equally important and interact between each other (see Additional File 15). We use the APEASE to retain applicability and practicality across diverse health settings/services.

The 80 domains agreed for inclusion in the behaviour change intervention, along with intervention functions and behaviour change techniques are presented in Additional File 16. COM-B domains included in the behaviour change intervention for each of the target levels (i.e. services, practitioners and patients) are presented in Table 4. The TIDieR Checklist [33] including the description of the behaviour change intervention is presented in Additional File 17.

**Table 4:** COM-B domains included in the behaviour change intervention targeted at services, practitioner and patient level

		Target levels		
COM-B DOMAINS		Services	Practitioners	Patients
**CAPABILITY**	**Physical capability**			
	**Psychological capability**	• Provide knowledge on procedures and guidelines to deliver psychological interventions remotely • Boost practitioners’ telephone skills	• Provide knowledge about the origins, drivers, and processes of telephone treatment • Develop telephone skills to enable a good therapeutic relationship, improve patient engagement, deliver patient-centred care, and effectively deliver treatment without visual aids and non-verbal cues	• Improve knowledge on psychological treatments (e.g. counselling, cognitive behavioural therapy, guided-self-help) and its different modes of delivery (e.g. face-to-face, telephone, group, online)
**OPPORTUNITY**	**Physical opportunity**	• Ensure practitioners are working in a comfortable and confidential environment • Ensure resources needed for telephone delivery are available		
	**Social opportunity**	• Provide regular assessment and monitoring of telephone performance in service • Promote learning and collaborative work across practitioners	• Provide assessment and monitoring of telephone performance during training and clinical practice	
**MOTIVATION**	**Automatic motivation**		Identify feelings related to working by telephone, and discuss feelings of being undervalued	
	**Reflective motivation**	• Promote reflective practice (e.g. telephone performance, beliefs and emotions related to working remotely, professional role expectations/challenges)	• Challenge negative beliefs associated to telephone treatment (e.g. telephone is a lower version of treatment)	

## Discussion

Behaviour change is more likely to be achieved if decisions as to intervention content are made following systematic guidelines such as those proposed in the Behaviour Change Wheel approach [1]. However, frameworks and guidelines offer a foundation for intervention development but require additional elucidation at each stage of the process, and our study provides a worked example of how to navigate through those challenges balancing theory, practicalities, resources and time.

The present research thus illuminates the sometimes-opaque transition from a research evidence base to a behaviour change intervention aiming to improve quality and engagement of telephone treatment in mental health services. We articulated three phases, namely, evidence synthesis, stakeholder involvement and decision-making in the development of our intervention. This research makes important contributions to the literature by providing solutions for addressing uncertainties about where and how judgement might be needed while developing an evidence-based behaviour change intervention. It additionally provides clear direction on how to optimise implementation of a behaviour change intervention to improve psychological interventions delivered by telephone in a complex system, aligning with Moore et al.’s [34] view of interventions as events within complex social systems, mental health policy and service improvement. The following discussion focuses on exploring the challenges we faced at each of our three proposed phases (i.e. evidence synthesis, stakeholder involvement and decision-making), and how we addressed them; in addition, the clinical implications of the intervention we developed are highlighted.

### Phase 1: Evidence synthesis

Questions may arise when deciding on the best approach to integrating and synthesising data from multiple sources that have used different methods of theory-informed analysis, such as if the use of a framework might be useful or if it would be beneficial to involve people from different disciplinary/professional perspectives closely related (or not) to the evidence.

The gaps between theory and practice interfere with the implementation of an evidence-based practice approach, and this has led to the development of different theoretical frameworks/models by which implementation might be more likely to succeed. However, choosing the most appropriate framework/model to design an intervention is challenging. We have chosen to use the Behaviour Change Wheel [1] approach, in which the COM-B and the TDF [20] models are at the core of it, because compared to other viable frameworks [32, 35], this offers additional advantages. Specifically, the COM-B and the TDF frameworks are closely related and offer additional, targeted and easily translatable knowledge on the determinants of behaviour change processes for other phases of the design and development of an evidence-based intervention.

The corpus of the evidence in which our intervention is grounded included multiple sources of data, highlighting that no individual source will be complete or perfect and the integration of different sources of data provides a deeper and integrated understanding of the phenomenon under investigation. It is important to highlight that a meta-analysis offers robust quality assessment on a topic (i.e. effectiveness). However, the design of an evidence-based intervention needs to be further informed by a comprehensive understanding on barriers and enablers that in our worked example are related to improve the quality of psychological treatment delivered by telephone and aid its implementation in clinical practice. Consequently, conducting several studies towards this end was key and we benefited from synthesising relevant evidence that would not have been available in a meta-analysis. We gained a deep understanding informed by the COM-B and TDF of challenges faced on the use of telephone delivery, and identified key behaviours that need to change at service, practitioner, patient and community levels to implement effectively remotely administered care in clinical practice. The involvement of a multidisciplinary programme team at early stages of the design of an intervention was crucial to bring together the expertise and skills of different professionals. In addition, from a pragmatic point of view, the evidence synthesis was performed in a single day.

### Phase 2: Stakeholder involvement

One of the weaknesses in the implementation of the Behavioural Change Wheel [1] is the lack of involvement of stakeholders [5], although the situation is changing and authors are increasingly incorporating stakeholder perspectives in their intervention development [36–38]. In our worked example, we incorporated stakeholder perspectives and provided solutions to potential issues such as how to include diversity and equity of voice and power across stakeholder groups and how to capture their views in a systematic and auditable way.

The involvement of stakeholders in health and social care is highly encouraged [4]. The contributions from stakeholders in the development of a behaviour change intervention, such as the one described in our worked example, are key to ensure that views from those who will use the intervention in the real world have been included to maximise the likelihood of success at implementation. For our intervention, key stakeholder groups included patients, practitioners and key informants (e.g. service managers, clinical leads) and we gathered their opinions on the design of the behaviour change intervention using the RAND/UCLA method [31]. This method, differently to other consensus methods, provides opportunities to identify and discuss areas of agreement within and across groups of participants instead of forcing consensus. In addition, holding meetings with each group of stakeholders independently and considering the ratings provided by group separately, ensured equal opportunity and influence to inform on the intervention development process, i.e. behaviour change intervention domains endorsed as essential by each of the groups were moved into the next phase of the process. The use of a systematic method such as the RAND/UCLA provides transparency into the development of an intervention and makes the process easy to audit.

### Phase 3: Decision-making

There is a gap in guidelines on the involvement of stakeholders [5] in the implementation of the behavioural change wheel [1]. The inclusion of stakeholders on the design of an intervention has its own challenges, such as how to resolve contradictions across stakeholder’s perspectives and contradictions between their views and the evidence. In addition, other issues related to the last phase of the development of the intervention were related to the identification of the behaviour change domains to be included in the final intervention; once identified, how to maximise the likelihood of success and effectiveness of the intervention into clinical practice and how to ensure evaluation and replicability of the designed intervention need to be determined.

In our worked example, domains endorsed as essential by a majority of stakeholder groups were included in the behaviour change intervention; no stakeholder group views were prioritised over others. The close interaction between a multidisciplinary programme team and groups of stakeholders such as in our example offers a feedback loop at multiple stages of the design of the intervention, maximising the potential of success and effectiveness of the intervention when implemented in clinical practice. In addition, a systematic and auditable intervention such as the one we described facilitates data to be cross-matched and the time from evaluation to refinement is likely to be more efficient and focused. Furthermore, the description of the process to develop an intervention is very much important to facilitate process evaluation [39]. Process evaluation is important to know not only if the intervention works but also to understand *how* it works to produce outcomes (i.e. identifying which aspects of the intervention are important, how different elements of an intervention work together, and how and intervention can be implemented in a specific context) [39]. Process evaluation would ultimately provide evidence to evaluate for intended/unintended consequences and actual effect of decisions taken in the development phase. Evidence on intervention development such as the one provided in our worked example would contribute to know how frameworks such as the Behaviour Change Wheel are being used and ultimately provide information to expand understanding on best ways to translate research into practice.

In our worked example, the developed intervention included multiple components to prompt behaviour change at service, practitioner and patient levels to improve quality of telephone-based service delivery models. In brief, the intervention focuses on:

1. At a service level: develop robust guidelines and standardise procedures for telephone delivery, adjust professional working environment and increase resources, provide clinical support for remote delivery and opportunities for professional development.
2. At a practitioner level: improve knowledge on the origins, drivers and processes for telephone delivery, develop practitioners telephone skills and dilute negative preconceptions about telephone treatment.
3. At a patient level: increase awareness on different psychological treatments (e.g. counselling, cognitive behavioural therapy, guided-self-help) and the different modes of delivery (e.g. telephone, group), and challenge beliefs of remote delivery being an inferior quality of care option compared to face-to-face.

### Strengths and limitations

Our work provides a transparent and systematic basis for future research aimed at developing behaviour change interventions. The development of an intervention using a systematic approach such as the behaviour change wheel offers guidelines for an effective evaluation. In addition, the use of a systematic method such as the RAND/UCLA to involve views from stakeholder groups allows equal opportunity/power, for their voices to be included on the design of the intervention. A limitation of our research is the absence of training for stakeholders in research methods and the behaviour change wheel to enable their participation at each of the development phases of the intervention; a preferable scenario if resources and time are available.

### Next steps

Following the Medical Research Council guidance [40], the behaviour change intervention described in this study will be piloted in a feasibility study and then based on the results, a decision will be made regarding conducting a randomised controlled trial (RCT). The RCT aims to evaluate the effectiveness of the behaviour change intervention developed to improve quality of delivery and engagement of patients on telephone treatment for common mental health difficulties in mental health services (for more details https://fundingawards.nihr.ac.uk/award/RP-PG-1016-20010; https://sites.manchester.ac.uk/equity/). A complete version of the TIDIeR checklist [33] will be made available following implementation of the intervention at the feasibility/RCT.

## Conclusion

This study provides a worked example on how to overcome challenges and make judgements while using a framework approach such as the Behaviour Change Wheel. We described three phases, moving from the evidence to the intervention involving stakeholders in the design process. We believe this represents a transparent and systematic framework for developing behaviour change interventions in any setting.

## Supplementary Information

**Additional file 1.** Evidence synthesis: Corpus of evidence on our worked-example

**Additional file 2.** Evidence synthesis: Description of attendees and procedures

**Additional file 3.** Matrix used for evidence synthesis of findings using the COM-B model

**Additional file 4.** Examples of the tasks conducted at the synthesis phase

**Additional file 5.** COM-B domain items and its corresponding TDF domains included in the proposed behavioural change intervention to be rated by stakeholder groups

**Additional file 6.** Stakeholder involvement: Recruitment and RAND/UCLA method

**Additional file 7.** Demographic characteristics for the stakeholder groups

**Additional file 8.** Post-Meeting Experiences Questionnaire for each of the three stakeholder groups

**Additional file 9.** Questionnaire to gather information from practitioners and key informants views at stakeholder meetings about the portion of the intervention to be targeted with practitioners (i.e. telephone training)

**Additional file 10.** Domains ratings from Round 1 and Round 2 per stakeholder group

**Additional file 11.** Minutes from moderated discussions from domains rated as “Not important” (i.e. Median <7) at Round 1

**Additional file 12 a** Domains rated at Round 3 as “Essential” (i.e. median between 7 and 9) by patients, practitioners or key informants. **b** Domains rated at Round 3 as “Not essential” (i.e. median <7) by patients, practitioners or key informants

**Additional file 13.** Post-Meeting Experiences for each of the three stakeholder groups

**Additional file 14.** Decision-Making: Description of attendees and procedures

**Additional file 15.** Recommendations: Target levels for the behaviour change intervention

**Additional file 16.** Intervention function and behaviour change techniques identified using the Behaviour Change Taxonomy (Version 1) for each domain included in the behaviour change intervention

**Additional file 17.** The Template for Intervention Description and Replication (TIDieR) Checklist

## Data Availability

The datasets supporting the conclusions of this article are included within the article (and its additional files).

## Abbreviations

APEASE: Acceptability, Practicability, Effectiveness/cost-effectiveness, Affordability, Safety/side-effects and Equity
COM-B: Capabilities, Oprtunities, and Motivations model of Behaviour
EQUITy: Enhancing the quality of psychological interventions delivered by Telephone
NIHR: National Institute for Health Research
RCT: Randomised Controlled Trial
TIDieR: Template for Intervention Description and Replication
IAPT: Increasing access to psychological therapies
LEAP: Lived Experience Advisory Panel
NICE: National Institute for Health and Care Excellence
NPT: Normalization Process Theory
RAND/UCLA: RAND Health/University of California-Los Angeles Appropriateness Methodology
TDF: Theoretical Domains Framework
TFA: Theoretical Framework of Acceptability
UK: United Kingdom
WHO: World Health Organization
IAPT: Increasing access to psychological therapies
LEAP: Lived Experience Advisory Panel
NICE: National Institute for Health and Care Excellence
NPT: Normalization Process Theory
RAND/UCLA: RAND Health/University of California-Los Angeles Appropriateness Methodology
TDF: Theoretical Domains Framework
TFA: Theoretical Framework of Acceptability
UK: United Kingdom
WHO: World Health Organization
