# Supplementary material for: Enhancing the Behaviour Change Wheel with synthesis, stakeholder involvement and decision-making: a case example using the ‘Enhancing the Quality of Psychological Interventions Delivered by Telephone’ (EQUITy) research programme
Source: Implement Sci. 2021 May 14;16:53. doi: 10.1186/s13012-021-01122-2 (PMC8120925; doi:10.1186/s13012-021-01122-2)
Supplement: Supplementary file 1 — Additional file 1. Evidence synthesis: Corpus of evidence on our worked-example [file 13012_2021_1122_MOESM1_ESM.docx]

**Additional File 1.** Evidence synthesis: Corpus of evidence on our worked-example

| ***Evidence collected as part of the research programme*** |
| --- |
| - Five primary qualitative studies   1) The aim of the study was to explore the views of patients with anxiety and/or depression on psychological interventions delivered by telephone and identify targets for change to improve engagement with this mode of delivery. Twenty-eight patients were interviewed. Data were analysed using the Theoretical Domains Framework (TDF)^[[1]](#footnote-1)^ and the Theoretical Framework of Acceptability (TFA)^[[2]](#footnote-2)^. The TDF is a conceptual base for understanding the determinants of behaviour change processes, identify implementation problems and inform the design of interventions. The TFA provides an objective, evidence-based assessment and evaluation of the acceptability of a given intervention. This research study is published^[[3]](#footnote-3)^.  2) The aim of the study was to explore the views of Step 2 practitioners on delivering low intensity psychological interventions by telephone (i.e. guided-self-help) and identify factors to improve quality of care and facilitate implementation of its use. Thirty-four Step 2 practitioners were interviewed. Data were analysed using the Theoretical Domains Framework (TDF)^1^. This research study is published^[[4]](#footnote-4)^.  3) The aim of the study was to explore the views of Step 3 practitioners on psychological interventions delivered by telephone. Eleven Step 3 practitioners were interviewed. Data were analysed using the Theoretical Domains Framework (TDF)^1^. This study is under preparation for journal submission.  4) The aim of the study was to explore the views of key informants (e.g. service managers, clinical leads, national decision-makers) and identify the challenges on the implementation of psychological treatment by telephone in mental health services. Twenty-one key informants participated in the study, 16 were interviewed individually and 5 on a focus group. Data were analysed using the Normalisation Process Theory (NPT)^[[5]](#footnote-5)^. The NPT framework allows exploration of factors that promote or inhibit implementation on clinical practice. This research study is published^[[6]](#footnote-6)^.  5) The aim of the study was to understand patient-practitioner communication on psychological interventions delivered over the telephone by Step 2 practitioners. One hundred and twenty three recordings of telephone sessions (i.e. assessment and treatment) were analysed using conversational analysis (CA)^[[7]](#footnote-7)^. This research study ispublished^[[8]](#footnote-8)^.   - Two secondary studies     1) A systematic rapid review investigating the effectiveness of training interventions to deliver therapy for common mental health difficulties (e.g. anxiety, depression) over the telephone in primary care and community settings. Four databases (i.e. CINAHL Plus, Web of Science, PsycInfo, PubMed) were included in the search and 4751 references identified for screening. After removing duplicates, 3253 studies screened by title and abstract, from which 620 studies were assessed for full-text eligibility. At the point of the meeting, 141 studies were included for data extraction and details from those were reported.  2) A literature mapping exercise exploring the existing curricula of university training courses (N=8) for Step 2 practitioners and telephone training delivered by mental health services (N=9). From the 8 university training courses that provided information, 7 reported delivery of telephone specific training; and from the 9 mental health services that provided information, 2 delivered telephone specific training. |
| ***Evidence from previous research*** |
| Examples of evidence from previous research that were reviewed:   - Delivering case management by telephone have equivalent effectiveness on depressive symptoms compared to face-to-face when implemented as part of a collaborative care intervention.^[[9]](#footnote-9)^ - Evidence-based psychological therapies (e.g. cognitive behavioural therapy) have shown promising results in reducing symptoms of depression and/or anxiety in adults when delivered over the telephone.^[[10]](#footnote-10)^ - Professionals perceived telephone delivery as a high-risk option. Experiential knowledge has been found to be the key to identify advantages and value of using the telephone (e.g. improving access). Existing protocols should be amended to highlight new methods of exchanging information and further clarification of the role and scope of using the telephone is needed to increase uptake and facilitate its implementation.^[[11]](#footnote-11)^ - Psychological interventions delivered using remote communication technologies have the potential to overcome barriers to conventional face-to-face delivery, such as physical, psychological and geographical constrains. However, further good quality research is needed to confirm its effectiveness in reducing symptoms of anxiety and depression.^[[12]](#footnote-12)^ - Majority of users receiving cognitive behavioural therapy delivered by telephone perceived the telephone as an acceptable context for therapeutic interaction. In addition, most users adapted and responded to telephone delivery and prioritised the accessibility and availability of services.^[[13]](#footnote-13)^ - Advantages of psychological therapy delivered by telephone among patients with chronic widespread pain included improving access and enabling change ‘in situ’ (i.e. delivering therapy directly into the context where behavioural change and/or pain management needed to occur). Furthermore, remote communication technologies is suggested to overcome stigma and patient resistance for psychological therapy.^[[14]](#footnote-14)^ - Cognitive behavioural therapy delivered by telephone was found to be acceptable, suitable and effective for patients with chronic widespread pain. The majority of patients attributed positive changes to the intervention such as feelings of empowerment and increased self-management.^[[15]](#footnote-15)^ - Low intensity interventions using a cognitive behavioural approach delivered by telephone in IAPT services have shown to be equally effective compared to face-to-face for anxiety symptoms, depression symptoms, work and social functioning in all people except for those with more severe illness.^[[16]](#footnote-16)^ - Evidence exploring interactional aspects of telephone and face-to-face psychological therapy highlighted no differences in relation to therapeutic alliance, disclosure, empathy, attentiveness or participation. However, wider implementation of telephone delivery remains a challenge due to ambivalence towards this mode of delivery among practitioners and patients.^[[17]](#footnote-17)^ - Mental health clinicians were apprehensive about telephone working prior to its delivery, but many reported positive experiences after conducting telephone assessments. Telephone assessments were reported to be structured, focused and comprehensive, and therapeutic rapport was able to be established. However, concerns persisted around whether risk assessments could be adequately conducted over the telephone.^[[18]](#footnote-18)^ - The clinical outcome of cognitive behaviour therapy delivered by telephone for treatment of obsessive compulsive disorder was equivalent to treatment delivered face-to-face and patients reported similarly high levels of satisfaction. Telephone sessions were 30 minutes shorter than face-to-face influencing costs and therapist time.^[[19]](#footnote-19)^ - Cognitive behavioural therapy delivered by telephone among patients with chronic widespread pain was associated with substantial, statistically significant, and sustained improvements (i.e. follow up at 9 and 24 months) in patient global health assessment.^[[20]](#footnote-20)^ - Cognitive behavioural therapy delivered by telephone did not show significant differences in client or therapist working alliance when compared to face-to-face.^[[21]](#footnote-21)^ - Cognitive behavioural therapy delivered by telephone for adolescents with obsessive compulsive disorder was equally effective compared to face-to-face. In addition, there were no significant differences in treatment credibility ratings provided by adolescents and by parents in the telephone group compared to the face-to-face group. Adolescents reported high levels of satisfaction with the help received.^[[22]](#footnote-22)^ |

Note. Step 2 practitioners are low intensity therapy workers (i.e. psychological well-being practitioners) trained in cognitive behavioural approaches for people with mild to moderate anxiety and depression and step 3 practitioners are high intensity therapy workers trained in different types of therapy (i.e. cognitive behavioural therapy, counselling, couple therapy, brief dynamic interpersonal therapy, interpersonal psychotherapy) for a range of mental health problems.

1. Cane J, O’Connor D, Michie S. Validation of the theoretical domains framework for use in behaviour change and implementation research. IS. 2012; 7: 37. [↑](#footnote-ref-1)
2. Sekhon M, Cartwright M, Francis JJ. Acceptability of healthcare interventions: an overview of reviews and development of a theoretical framework. BMC Health Serv Res. 2017; 17(1): 88. [↑](#footnote-ref-2)
3. Rushton K, Ardern K, Hopkin E, Welsh C, Gellatly J, Faija CL, et al. ‘I didn’t know what to expect’: Exploring patient perspectives to identify targets for change to improve telephone-delivered psychological interventions. BMC Psychiatry. 2020; 20:156. [↑](#footnote-ref-3)
4. Faija CL, Connell J, Welsh C, Ardern K, Hopkin E, Gellatly J, et al. What influences practitioners’ readiness to deliver psychological interventions by telephone? A qualitative study of behaviour change using the Theoretical Domains Framework. BMC Psychiatry. 2020, 20:371. [↑](#footnote-ref-4)
5. Murray E, Treweek S, Pope C, MacFarlane A, Ballini L, Dowrick C, et al. Normalisation process theory: a framework for developing, evaluating and implementing complex interventions. BMC Med. 2010; 8(1): 63. [↑](#footnote-ref-5)
6. Rushton K, Fraser C, Gellatly J, Brooks H, Bower P, Armitage CJ, et al. A case of misalignment: The perspectives of local and national decision-makers on the implantation of psychological treatment by telephone in the Improving Access to Psychological Therapies Service. BMC Health Serv Res. 2019; 20:36. [↑](#footnote-ref-6)
7. Jefferson G. Glossary of transcript symbols with an introduction. In G. H. Lerner (Ed). Conversation Analysis: Studies from the First Generation. (pp: 13-31). Amsterdam: John Benjamins. 2004 [↑](#footnote-ref-7)
8. Irvine A, Drew P, Bower P, Ardern K, Armitage, Barkham, et al. ‘So just to go through the options…’: Patient choice in the telephone delivery of the NHS Improving access to Psychological Therapies services.  Sociol Health Illn.2020,  <https://doi.org/10.1111/1467-9566.13182>. 2020, [↑](#footnote-ref-8)
9. Hudson JL, Bower P, Kontopantelis E, Bee P, Archer J, Clarke R, et al. Impact of telephone delivered case-management on the effectiveness of collaborative care for depression and anti-depressant use: A systematic review and meta-regression. PloS ONE. 2019; 14(6): e0217948. [↑](#footnote-ref-9)
10. Coughtrey AE, Pistrang N. The effectiveness of telephone-delivered psychological therapies for depression and anxiety: A systematic review. J Telemed Telecare. 2016; 24(2): 65-74. [↑](#footnote-ref-10)
11. Bee P, Lovell K, Airnes Z, Pruszynska A. Embedding telephone therapy in statutory mental health services: A qualitative, theory-driven analysis. BMC Psychiatry. 2016; 16:56. [↑](#footnote-ref-11)
12. Bee P, Lovell K, Bower P, Gilbody S, Richards D, Gask L, & Roach P. Psychotherapy mediated by remote communication technologies: A meta-analytic review. BMC Psychiatry. 2008; 8:60. [↑](#footnote-ref-12)
13. Bee P, Lovell K, Lidbetter N, Easton K, Gask L. You can’t get anything perfect: “User perspectives on the delivery of cognitive behavioural therapy by telephone.” Soc Sci Med. 2010; 71(7): 1308-1315. [↑](#footnote-ref-13)
14. Bee P, McBeth J, MacFarlane GJ, Lovell K. Managing chronic widespread pain in primary care: A qualitative study of patient perspectives and implications for treatment delivery. BMC Musculoskelet Disord. 2016; 17: 354 [↑](#footnote-ref-14)
15. Fraser C, Beasley M, Macfarlane G, Lovell K. Telephone cognitive behavioural therapy to prevent the development of chronic widespread pain: A qualitative study of patient perspectives and treatment acceptability. BMC Musculoskelet Disord. 2019; 20: 198. [↑](#footnote-ref-15)
16. Hammond GC, Croudace TJ, Radhakrishnan M, Lafortune L, Watson A, McMillan-Shileds F, Jones P. Comparative Effectiveness of Cognitive Therapies Delivered Face-to-Face or over the Telephone: An Observational Study Using Propensity Methods. Plos One. 2012; 7(9): e42916. [↑](#footnote-ref-16)
17. Irvine A, Drew P, Bower P, Brooks H, Gellatly J, Armitage CJ, Barkham M, McMillan D, Penny B. (2020). Are there interactional differences between telephone and face-to-face psychological therapy? A systematic review of comparative studies. J Affect Disord. 2020; 265: 120-131. [↑](#footnote-ref-17)
18. Jones EA, Bale HL, Morera T. A qualitative study of clinicians’ experiences and attitudes towards telephone triage mental health assessments. The Cognitive Behaviour Therapist. 2013; 6(e17): 1-14. [↑](#footnote-ref-18)
19. Lovell K, Cox D, Haddock G, Jones C, Raines D, Garvey R, Roberts C, Hadley S. Telephone administered cognitive behaviour therapy for treatment of obsessive compulsive disorder: Randomised controlled non-inferiority trial. BMJ. 2006; 333(7574): 883. [↑](#footnote-ref-19)
20. McBeth J, Prescott G, Scotland G, Lovell K, Keeley P, Hannaford P, McNamee P, Symmons DP, Woby S, Gkazinou C, Beasley M, Macfarlane GJ. Cognitive behavior therapy, exercise, or both for treating chronic widespread pain. Arch. Intern. Med. 2012; 172(1): 48-57. [↑](#footnote-ref-20)
21. Stiles-Shields C, Kwasny MJ, Cai X, Mohr D. Therapeutic Alliance in Face-to-face and Telephone-Administered Cognitive Behavioral Therapy. JCCP. 2014; 82(2): 349-354 [↑](#footnote-ref-21)
22. Turner CM, Mataix-Cols D, Lovell K, Krebs G, Lang K, Byford S, Heyman I. Telephone Cognitive-Behavioral Therapy for Adolescents With Obsessive-Compulsive Disorder: A Randomized Controlled Non-inferiority Trial. JAACAP. 2014; 53(12): 1298–1307. [↑](#footnote-ref-22)
