# Supplementary material for: Enhancing the Behaviour Change Wheel with synthesis, stakeholder involvement and decision-making: a case example using the ‘Enhancing the Quality of Psychological Interventions Delivered by Telephone’ (EQUITy) research programme
Source: Implement Sci. 2021 May 14;16:53. doi: 10.1186/s13012-021-01122-2 (PMC8120925; doi:10.1186/s13012-021-01122-2)
Supplement: Supplementary file 2 — Additional file 2. Evidence synthesis: Description of attendees and procedures [file 13012_2021_1122_MOESM2_ESM.docx]

**Additional File 2.** Evidence synthesis: Description of attendees and procedures

| ***Attendees*** |
| --- |
| All members of the programme team (N=27) were invited to take part in the synthesis day meeting and 16 attended. Attendees included clinicians (GPs, clinical psychologists, health psychologist, mental health professionals who had/were working in IAPT), academics and researchers with experience in mental health services and primary care settings, delivery and evaluation of behaviour change interventions and implementation science; and the patient and public involvement representative. |
| ***Procedure*** |
| **Before the meeting**  Two weeks prior to the synthesis day meeting, slides for a 5-minutes presentation along with brief single-page summaries of the key findings and recommendations for intervention development for each of the 7 studies (5 primary studies and the 2 secondary studies) conducted as part of the research programme were circulated to the programme team (N=27). Comments from programme team members were invited. This ensured all members of the programme team were fully briefed prior to the evidence synthesis meeting and those who were unable to attend were able to submit written comments to contribute to the process of intervention development.  **At the meeting**  The evidence synthesis meeting comprised four tasks:  1) Findings from the seven studies were presented by the lead researchers who invited questions for clarification from the group. For each presentation, attendees were asked to identify the key domains they thought should be included in the intervention and write them on post-it notes. At the end of each presentation, participants’ notes were collected. After all the presentations, contradictions, conflicts and/or ambiguities between datasets were discussed.  2) Attendees were divided into seven subgroups (1 per study) comprising people with different levels of expertise, different disciplinary/professional perspectives and different levels of involvement in the data collection/analysis to review the post-it notes. The subgroups were asked to conduct a preliminary synthesis by removing duplicates and/or replacing post-it notes with new summaries.  3) Attendees were asked to map the proposed domains of the intervention onto the COM-B model of behaviour change to ensure that the evidence-base was aligned with the theoretical underpinnings. The COM-B model recognises that behaviour is part of an interacting system involving capabilities (C), opportunities (O) and motivations (M), these are the key drivers of behaviour change and are at the core of the behaviour change wheel^[[1]](#footnote-1)^. Thus, following a primer on the COM-B model, the seven subgroups categorised the post-it notes as to whether they were addressing people’s capabilities (physical and psychological), opportunities (physical and social) or motivations (automatic and reflective). **Additional File 3** includes details of the COM-B matrix used as part of this task to synthesise the evidence from multiple studies. **Additional File 4** shows pictures of the tasks conducted during the meeting.    4) Attendees were divided into three subgroups (1 per each COM-B area) to summarise the identified domains related to each of the three COM-B areas to be included in the behaviour change intervention. Then, each subgroup reported to the larger group and there was opportunity to discuss capabilities, opportunities and motivations further, considering changes at patient, practitioner, service and community levels.  **After the meeting**  A cross-validation exercise was conducted by two members of the programme team (KL, PBe) in which previous knowledge and clinical expertise on the topic was reviewed to cross-check findings resulted from the evidence synthesis day. |

1. Michie S, Atkins L, West R. The Behaviour Change Wheel: A guide to designing interventions. Great Britain: Silverback Publishing; 2014. [↑](#footnote-ref-1)
