# Supplementary material for: Enhancing the Behaviour Change Wheel with synthesis, stakeholder involvement and decision-making: a case example using the ‘Enhancing the Quality of Psychological Interventions Delivered by Telephone’ (EQUITy) research programme
Source: Implement Sci. 2021 May 14;16:53. doi: 10.1186/s13012-021-01122-2 (PMC8120925; doi:10.1186/s13012-021-01122-2)
Supplement: Supplementary file 3 — Additional file 3. Matrix used for evidence synthesis of findings using the COM-B model [file 13012_2021_1122_MOESM3_ESM.docx]

**Additional File 3.** Matrix used for evidence synthesis of findings using the COM-B model

|  |  | **Study 1**  Patient perspectives | **Study 2**  Step 2 Practitioners perspectives | **Study 3**  Step 3 Practitioners perspectives | **Study 4**  Key informants perspectives | **Study 5**  Patient-practitioner communication | **Study 6** Systematic Review | **Study 7** Literature  Mapping |
| --- | --- | --- | --- | --- | --- | --- | --- | --- |
| **Capability** | **Physical**  **Capability** |  |  |  |  |  |  |  |
|  | **Psychological**  **Capability** |  |  |  |  |  |  |  |
| **Opportunity** | **Physical**  **Opportunity** |  |  |  |  |  |  |  |
|  | **Social**  **Opportunity** |  |  |  |  |  |  |  |
| **Motivation** | **Automatic Motivation** |  |  |  |  |  |  |  |
|  | **Reflective**  **Motivation** |  |  |  |  |  |  |  |
