# Supplementary material for: Enhancing the Behaviour Change Wheel with synthesis, stakeholder involvement and decision-making: a case example using the ‘Enhancing the Quality of Psychological Interventions Delivered by Telephone’ (EQUITy) research programme
Source: Implement Sci. 2021 May 14;16:53. doi: 10.1186/s13012-021-01122-2 (PMC8120925; doi:10.1186/s13012-021-01122-2)
Supplement: Supplementary file 4 — Additional file 4. Examples of the tasks conducted at the synthesis phase [file 13012_2021_1122_MOESM4_ESM.docx]

**Additional File 4.** Examples of the tasks conducted at the synthesis phase**^[[1]](#footnote-2)^**

1. **Categorising the evidence based on Capability, Opportunity, and Motivation (COM-B Model) to improve delivery by professionals of psychological interventions over the telephone**


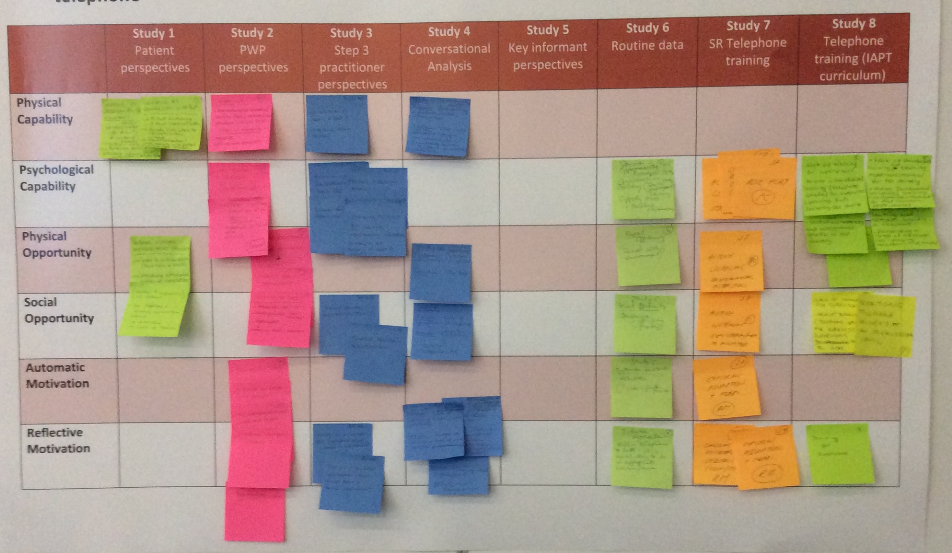


**b) Categorising the evidence based on Capability, Opportunity, and Motivation (COM-B Model) to improve engagement by patients with anxiety and/or depression to psychological interventions over the telephone**


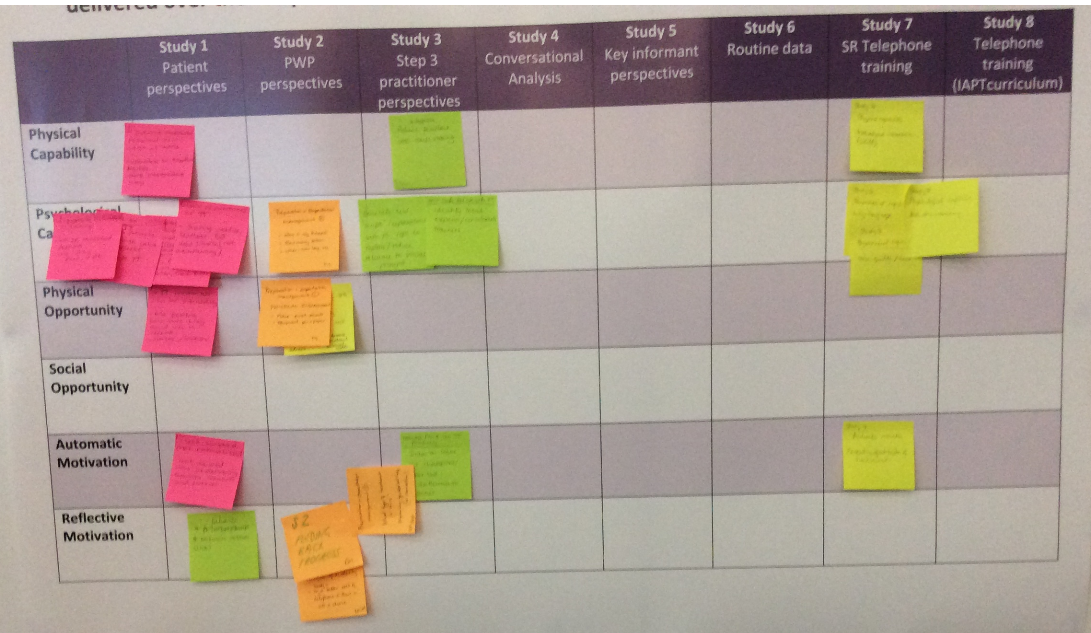


1. Note that findings from “study 6” were not included in the synthesis day because access to routine data were not granted in sufficient time [↑](#footnote-ref-2)
