# Supplementary material for: Enhancing the Behaviour Change Wheel with synthesis, stakeholder involvement and decision-making: a case example using the ‘Enhancing the Quality of Psychological Interventions Delivered by Telephone’ (EQUITy) research programme
Source: Implement Sci. 2021 May 14;16:53. doi: 10.1186/s13012-021-01122-2 (PMC8120925; doi:10.1186/s13012-021-01122-2)
Supplement: Supplementary file 6 — Additional file 6. Stakeholder involvement: Recruitment and RAND/UCLA method [file 13012_2021_1122_MOESM6_ESM.docx]

**Additional File 6.** Stakeholder involvement: Recruitment and RAND/UCLA method

| ***Ethics*** |
| --- |
| The stakeholder involvement phase (phase 2) of the behaviour change intervention development was approved by North West Greater Manchester West Research Ethics Committee (Ref: 18/NW/0372). Data collection from stakeholders took place between September and December 2019. |
| ***Recruitment*** |
| Stakeholders were recruited from UK NHS trusts, third sector organisations that were commissioned to deliver Improving Access to Psychological therapies (IAPT) services for people experiencing common mental health difficulties, Self-Help Groups, GP surgeries, Universities, and via intranet and social media.  Stakeholders were invited to take part in a group meeting to discuss a behaviour change intervention to improve engagement and quality of care of psychological interventions by telephone.  Stakeholder inclusion criteria:  -*Patients* were eligible to take part if they had received/were receiving/waiting to receive psychological treatment and/or support for anxiety and/or depression in any mode of delivery (face-to-face, group, telephone, on-line).  -*Practitioners* were eligible to take part if they were qualified or in training with current or future responsibility for delivering guided-self-help interventions over the telephone for common mental health difficulties in Improving Access to Psychological Therapies (IAPT) services.  -*Key informants* were eligible to take part if they had managerial responsibilities within mental health services, if they were involved in training practitioners delivering low-intensity psychological interventions (i.e. Step 2 practitioners), in the development and implementation of IAPT or NHS policy, in research and/or had clinical experience.  Potential participants received a participant information sheet and a consent to contact form. Those that were interested in taking part were asked to contact the designated researcher (CF) via email or to complete and return the ‘consent to contact form’ to the research team by email or post. Researchers were available to respond to any questions. All participants were asked to provide written informed consent and were reimbursed for their travel expenses. A gift voucher was given as a compensation and thank you for their time.  Twenty-two patients expressed an interest in taking part of whom seven actually attended; 30 practitioners expressed interest in taking part of whom 19 attended, and 29 key informants expressed interest in participating of whom 15 attended. Demographic characteristics for the stakeholder groups are included in **Additional File 7**. From the 19 practitioners, 52.6 % had supervisory responsibilities, 89.5 % had worked in mental health from 1 to 10 years, and 89.5 % had delivered psychological interventions by telephone. From the 15 key informants, 5 were service leads, 7 were national leads/influencers/policy makers, and 3 were academics; 80% of key informants had worked in mental health for over 10 years and 66.7 % had experienced delivering psychological interventions by telephone. All stakeholders completed a post-meeting questionnaire (**Additional File 8**), and Practitioners and Key Informants provided additional views regarding practicalities of the practitioner training (**Additional File9**). |
| ***RAND/UCLA Method*** |
| ***RAND/UCLA Adaptations***  The 93 domains from phase 1 were presented on a form to be rated developed by CF and revised by CA and PBe initially, and then by the wider programme team. A group of service users with experience of anxiety and/or depression or carers (N=6), who comprised the Lived Experience Advisory Panel (LEAP), additionally ensured the form was accessible and user-friendly for patients. In addition to extending the sampling beyond purely academic experts, we made changes to the standard RAND/UCLA wording of the terms ‘appropriateness’ and ‘necessity’ on the basis of advice from LEAP. The LEAP disagreed with the terms ‘appropriateness’ and ‘necessity’, and suggested replacing them with ‘importance’ and ‘essentiality’, respectively. It was pointed out that all the suggested domains to be rated by stakeholders must be ‘appropriate’ because they were identified from the evidence and that it was difficult to think in terms of more or less appropriate. They further argued that an intervention to enhance engagement and quality of psychological interventions delivered by telephone was very much needed, and advised that the word ‘essential’ would better capture the domains that should be addressed in the behaviour change intervention. Consequently, each of the 93 domains presented on the form were rated on a 9-point Likert scale ranging from 1 (Not important/Not essential) to 9 (Extremely important/Essential) (**Additional File 5**)**.**  ***RAND/UCLA Procedure***  Patients, practitioners and key informants participated in three separate daylong meetings, for which they were provided with identical materials. Consistent with the RAND/UCLA method, each of the three meetings had the same structure and comprised three rounds:  i) individual ratings of appropriateness (labelled ‘importance’ in our worked-example)  ii) moderated group ratings of appropriateness (labelled ‘importance’ in our worked-example),  iii) individual ratings of necessity (labelled ‘essential’ in our worked-example).  Participants made their ratings online via Nearpod [49], a platform for collating responses from multiple sources in real time, using their own or provided, internet-enabled devices. Participants received an ID number to maintain anonymity.  In Round 1, participants rated individually and anonymously each of the 93 domains for importance after which the data were exported to Excel. After Round 1, participants were provided with a paper copy of their personal ratings and the anonymised data of others. Consistent with Fitch et al.’s guidelines [33], domains with medians between 7 and 9 were considered ‘Extremely important’ and so not in need of further discussion.  Domains rated in Round 1 with a median lower than 7 (4 to 6 is ‘Moderately important’; 1 to 3 is ‘Not important’) were subject to moderated discussion involving the whole group after which the domains were rated again in terms of ‘importance’ in Round 2. A health psychologist with experience using this approach (CA) led the group discussion facilitating open conversation and encouraging participation from the group. Discussions that occurred during Round 2 were audio recorded with the consent of all participants.  In Round 3, participants were asked to rate all 93 domains on ‘essentiality’. Again, domains with a median rating of 7 to 9 were considered ‘Essential’ and to be included in the behaviour change intervention.  Ratings from all rounds were analysed using descriptive statistics of frequencies and medians. As noted above, medians of 7 or above were considered critical cut-offs.  Following the 3 rounds of rating, practitioners and key informants were asked to complete a 5-item questionnaire that is was designed to assess opinions about the portion of the intervention  that would be targeted at practitioners (e.g. length of training, training methods) (**Additional File 8**).  At the end of the meeting, all stakeholders were asked to complete a demographic questionnaire and a questionnaire gathering details of their experiences of taking part, which was adapted from the RAND/UCLA template form (Fitch et al., 2001, p. 41) (**Additional File9**). |
