# Supplementary material for: Enhancing the Behaviour Change Wheel with synthesis, stakeholder involvement and decision-making: a case example using the ‘Enhancing the Quality of Psychological Interventions Delivered by Telephone’ (EQUITy) research programme
Source: Implement Sci. 2021 May 14;16:53. doi: 10.1186/s13012-021-01122-2 (PMC8120925; doi:10.1186/s13012-021-01122-2)
Supplement: Supplementary file 7 — Additional file 7. Demographic characteristics for the stakeholder groups [file 13012_2021_1122_MOESM7_ESM.docx]

**Additional File 7.** Demographic characteristics for the stakeholder groups

|  | ***Patients***  **(N=7)** | ***Practitioners***  **(N=19)** | ***Key Informants***  **(N=15)** |
| --- | --- | --- | --- |
| **Age** | **M, SD, Range** | **M, SD, Range** | **M, SD, Range** |
|  | M=41.4, SD=17.02,  Range 18-62 | M=33.63, SD=9.06,  Range 23-53 | M=41.71, SD=8.84,  Range 30-55  (1 missing value) |
|  |  |  |  |
| **Gender** | **Frequencies (%)** | **Frequencies (%)** | **Frequencies (%)** |
| Male | 3 (42.9) | 4 (21.1) | 3 (20.0) |
| Female | 4 (57.1) | 15 (78.9) | 12 (80.0) |
| **Ethnicity** |  |  |  |
| White | 6 (85.7) | 18 (94.8) | 15 (100.0) |
| Other | 1 (14.3) | 1 (5.3) |  |
| **Highest Educational Qualification** |  |  |  |
| Pre-higher education | 4 (57.2) |  |  |
| Undergraduate education | 3 (42.9) | 3 (15.8) |  |
| Postgraduate education |  | 16 (84.3) | 14 (93.3) |
| Other |  |  | 1 (6.7) |
| **Employment status** |  |  |  |
| Employed |  | 19 (100.0) | 15 (100.0) |
| Not employed | 7 (100.0) |  |  |
