# Supplementary material for: Enhancing the Behaviour Change Wheel with synthesis, stakeholder involvement and decision-making: a case example using the ‘Enhancing the Quality of Psychological Interventions Delivered by Telephone’ (EQUITy) research programme
Source: Implement Sci. 2021 May 14;16:53. doi: 10.1186/s13012-021-01122-2 (PMC8120925; doi:10.1186/s13012-021-01122-2)
Supplement: Supplementary file 8 — Additional file 8. Post-Meeting Experiences Questionnaire for each of the three stakeholder groups [file 13012_2021_1122_MOESM8_ESM.docx]

**Additional File8.** Post-Meeting Experiences Questionnaire for each of the three stakeholder groups

Thank you for participating in our EQUITY programme, which aims to improve engagement and quality of psychological interventions delivered by telephone. As a final favour, we would appreciate your completing this questionnaire about your experience as a participant. For each item, please circle the appropriate number or fill in the blank. If you have any additional comments or suggestions, please note them at the bottom of the page.

| **FIRST ROUND RATINGS (individual ratings)** | ***Patients***  **(N=7)** | ***Practitioners***  **(N=19)** | ***Key Informants***  **(N=12*)** |
| --- | --- | --- | --- |
| **How easy did you find the task?** | **Frequencies (%)** | **Frequencies (%)** | **Frequencies (%)** |
| Not at all | 1 (14.3) | 1 (5.3) |  |
| A little | 1 (14.3) | 1 (5.3) |  |
| Somewhat | 2 (28.6) | 6 (31.6) | 7 (58.3) |
| Very much so | 3 (42.9) | 11 (57.9) | 5 (41.7) |
| **How difficult did you find the task?** |  |  |  |
| Not at all | 4 (57.1) | 13 (68.4) | 6 (50.0) |
| A little | 2 (28.6) | 5 (26.3) | 4 (33.3) |
| Somewhat |  | 1 (5.3) | 2 (16.7) |
| Very much so | 1 (14.3) |  |  |
| **How clear were the instructions?** |  |  |  |
| Not at all |  | 1 (5.3) |  |
| A little | 1 (14.3) | 1 (5.3) |  |
| Somewhat | 1 (14.3) | 5 (26.3) | 4 (33.3) |
| Very much so | 5 (71.4) | 12 (63.2) | 8 (66.7) |
| **How inconsistent do you believe you were? (due to effects of fatigue, memory, format of instrument, etc)** |  |  |  |
| Not at all | 2 (28.6) | 5 (26.3) | 2 (16.7) |
| A little | 3 (42.9) | 9 (47.4) | 7 (58.3) |
| Somewhat | 2 (28.6) | 5 (26.3) | 3 (25.0) |
| Very much so |  |  |  |
| **SECOND ROUND RATINGS (group ratings)** |  |  |  |
| **How effective did you think the group discussion was?** |  |  |  |
| Not at all |  |  |  |
| A little | 1 (14.3) | 1 (5.3) |  |
| Somewhat | 2 (28.6) | 9 (47.4) | 3 (25.0) |
| Very much so | 4 (57.1) | 9 (47.4) | 9 (75.0) |
| **How informative was the discussion?** |  |  |  |
| Not at all | 1 (14.3) |  |  |
| A little |  | 1 (5.3) |  |
| Somewhat | 1 (14.3) | 12 (63.2) | 1 (8.3) |
| Very much so | 5 (71.4) | 6 (31.6) | 11 (91.7) |
| **How argumentative was the discussion?** |  |  |  |
| Not at all | 2 (28.6) | 14 (73.7) | 8 (66.7) |
| A little | 3 (42.9) | 4 (21.1) | 4 (33.3) |
| Somewhat | 1 (14.3) |  |  |
| Very much so | 1 (14.3) | 1 (5.3) |  |
| **How much did the feedback from the first round ratings influence your second round ratings?** |  |  |  |
| Not at all |  |  | 1 (8.3) |
| A little | 4 (57.1) | 10 (52.6) | 4 (33.3) |
| Somewhat | 3 (42.9) | 7 (36.8) | 7 (58.3) |
| Very much so |  | 2 (10.5) |  |
| **How much did the discussion influence your second round ratings?** |  |  |  |
| Not at all |  |  |  |
| A little | 4 (57.1) | 11 (57.9) | 4 (33.3) |
| Somewhat | 3 (42.9) | 7 (36.8) | 8 (66.7) |
| Very much so |  | 1 (5.3) |  |
| **OVERALL IMPRESSIONS OF YOUR EXPERIENCE** |  |  |  |
| **How much do you think your own ratings will influence the final intervention?** |  |  |  |
| Not at all |  |  |  |
| A little | 3 (42.9) | 7 (36.8) | 6 (50.0) |
| Somewhat | 3 (42.9) | 12 (63.2) | 6 (50.0) |
| Very much so | 1 (14.3) |  |  |
| **How much do you think the group ratings will influence the final intervention?** |  |  |  |
| Not at all |  |  |  |
| A little | 1 (14.3) | 4 (21.1) | 3 (25.0) |
| Somewhat | 2 (28.6) | 6 (31.6) | 4 (33.3) |
| Very much so | 4 (57.1) | 9 (47.4) | 5 (41.7) |
| **How much do you believe that this process can lead to a set of recommendations to improve the quality of therapy delivered over the telephone?** |  |  |  |
| Not at all |  |  |  |
| A little |  | 1 (5.3) | 1 (8.3) |
| Somewhat | 2 (28.6) | 9 (47.4) | 5 (41.7) |
| Very much so | 5 (71.4) | 9 (47.4) | 6 (50.0) |
| **How much do you believe that this process can lead to a set of recommendations to improve patient engagement with therapy delivered over the telephone?** |  |  |  |
| Not at all |  |  |  |
| A little | 1 (14.3) | 1 (5.3) | 1 (8.3) |
| Somewhat | 4 (57.1) | 12 (63.2) | 6 (50.0) |
| Very much so | 2 (28.6) | 6 (31.6) | 5 (41.7) |
| **How satisfying did you find your participation on this meeting?** |  |  |  |
| Not at all |  |  |  |
| A little | 1 (14.3) | 2 (10.5) |  |
| Somewhat | 1 (14.3) | 12 (63.2) | 4 (33.3) |
| Very much so | 5 (71.4) | 6 (31.6) | 8 (66.7) |
| **How did your participation on this meeting compare with your expectations?** |  |  |  |
| Much worse |  |  |  |
| Worse |  |  |  |
| On a par | 2 (28.6) | 6 (31.6) | 8 (66.7) |
| Better | 2 (28.6) | 9 (47.4) | 4 (33.3) |
| Much better | 3 (42.9) | 4 (21.1) |  |

**Note:** *****Data from three key informants were missing.

**Comments:**______________________________________________________________________________________________________________________________________________________________________________________________________________________________________________________________________________________________________________________________
