# Supplementary material for: Enhancing the Behaviour Change Wheel with synthesis, stakeholder involvement and decision-making: a case example using the ‘Enhancing the Quality of Psychological Interventions Delivered by Telephone’ (EQUITy) research programme
Source: Implement Sci. 2021 May 14;16:53. doi: 10.1186/s13012-021-01122-2 (PMC8120925; doi:10.1186/s13012-021-01122-2)
Supplement: Supplementary file 9 — Additional file 9. Questionnaire to gather information from practitioners and key informants views at stakeholder meetings about the portion of the intervention to be targeted with practitioners (i.e. telephone training) [file 13012_2021_1122_MOESM9_ESM.docx]

**Additional File 9.** Questionnaire to gather information from practitioners and key informants views at stakeholder meetings about the portion of the intervention to be targeted with practitioners (i.e. telephone training)

| **1. How long should the telephone training be?** | Days___ // Hours ____ | |
| --- | --- | --- |
| **2. Which of the following training methods should we use?** Please choose up to five and rank in order of importance (1=most important, 5=Less important) |  | |
|  | **Tick** | **Rank** |
| a. Traditional instructive teaching |  |  |
| b. Traditional instructive teaching enhanced with clinical examples/demonstrations (audios of telephone treatment sessions, observing a telephone role-playing between trainers) |  |  |
| c. Telephone treatment role-playing between trainees (Patient & Practitioner role) |  |  |
| d. Group discussion |  |  |
| e. Online |  |  |
| f. Use of an established scale to assess whether the treatment delivered over the telephone by your Practitioner meets standards of good quality delivery |  |  |
| g. Use of audio-recordings during training to discuss what went well and what could be improved – Use audios to promote practitioner self-assessment of treatment delivered over the telephone (reflective learning) |  |  |
| h. Directly listening to telephone treatment sessions delivered by a Practitioner experienced in telephone treatment/Direct observation of telephone calls (shadowing) |  |  |
| **3. Who should attend the telephone training day(s)?** |  | |
| **4. Who should be delivering the training day(s)?** |  | |
| **5. Should the training provide points to go towards their professional development?** |  | |
