# Supplementary material for: Enhancing the Behaviour Change Wheel with synthesis, stakeholder involvement and decision-making: a case example using the ‘Enhancing the Quality of Psychological Interventions Delivered by Telephone’ (EQUITy) research programme
Source: Implement Sci. 2021 May 14;16:53. doi: 10.1186/s13012-021-01122-2 (PMC8120925; doi:10.1186/s13012-021-01122-2)
Supplement: Supplementary file 10 — Additional file 10. Domains ratings from Round 1 and Round 2 per stakeholder group [file 13012_2021_1122_MOESM10_ESM.docx]

**Additional File10.** Domains ratings from Round 1 and Round 2 per stakeholder group

| **Section A:** We are interested in what you think we can do to: (a) improve delivery by practitioners of psychological interventions over the telephone for patients with anxiety and/or depression, (b) improve engagement by patients with psychological interventions delivered over the telephone | **Patients**  **(N=7)** | | **Practitioners**  **(N=19)** | | **Key Informants**  **(N=15)** | |
| --- | --- | --- | --- | --- | --- | --- |
|  | **Round 1** | **Round 2** | **Round 1** | **Round 2** | **Round 1** | **Round 2** |
| **KNOWLEDGE: This section looks at what you would like to know in relation to psychological treatment delivered over the telephone and what do you think your practitioner should know before delivering psychological treatment over the telephone** |  |  |  |  |  |  |
| 1. How important is it to you as a patient to know about the psychological treatment that you will be receiving over the telephone? | **9** |  | **9** |  | **9** |  |
| **How important is it to you as a patient…** |  |  |  |  |  |  |
| 2. To know that telephone appointments are as formal and important as face-to-face? | **8** |  | **9** |  | **9** |  |
| 3. To know that the missed appointment/ discharge rules for treatment delivered over the telephone are the same as for face-to-face treatment? | **9** |  | **9** |  | **9** |  |
| 4. To know that treatment over the telephone is being delivered by the same qualified practitioners that deliver face-to-face treatments? | **7** |  | **9** |  | **8** |  |
| 5. To know that you should answer your session phone calls in a private, quiet and confidential place | **8** |  | **9** |  | **9** |  |
| 6. To have some information about your practitioner such as written information about their experience (a bio) and a picture of her/him? | **7** |  | 3 | 2 | 4 | 2 |
| 7. To know that your practitioner might be typing notes into the computer during the telephone session? | 6 | 6 | 6 | **8** | **7** |  |
| 8. How important is it to you as a patient that your practitioner knows about the delivery of psychological treatment over the telephone? | **9** |  | **8** |  | **9** |  |
| 9. That your practitioner knows about patient experiences of receiving treatment over the telephone? | **9** |  | **7** |  | **8** |  |
| 10. That your practitioner knows about other practitioner experiences of delivering treatment over the telephone? | **8** |  | **7** |  | **7** |  |
| 11. That your practitioner knows how successful/useful treatment is when delivered over the telephone in comparison to when it is delivered face-to-face? | **7** |  | **8** |  | **9** |  |
| 12. That your practitioner knows about specific guidelines to deliver telephone treatment? | **9** |  | **7** |  | **8** |  |
| **SKILLS: This section looks at what skills do you think are important to help you engage with treatment delivered over the telephone.** |  |  |  |  |  |  |
| 13. How important is it to you that your practitioner develops/adapts her or his skills to deliver treatment over the telephone? | **9** |  | **9** |  | **9** |  |
| 14. Practitioner should have verbal communication skills to suit treatment delivered over the telephone (e.g. using verbal skills to show they understand how you feel and that they care about you) | **9** |  | **9** |  | **9** |  |
| 15. Practitioner should develop skills to adjust their tone of voice over the telephone | **8** |  | **7** |  | **8** |  |
| 16. Practitioner should develop skills to use and deal with silences when talking over the telephone and recognise signals to know when to talk and when not to talk | **9** |  | **8** |  | **8** |  |
| 17. Practitioner should develop skills to create a feeling/sense of a ‘therapeutic space’ and a ‘safe space to talk’ when delivering treatment over the telephone | **9** |  | **8** |  | **9** |  |
| 18. Practitioner should develop skills to build up a good relationship with you over the telephone | **9** |  | **9** |  | **9** |  |
| 19. Practitioner should pay attention to what you are saying and give signals to let you know they are listening to you carefully when you are receiving treatment over the telephone | **9** |  | **9** |  | **9** |  |
| 20. Practitioner should develop skills to personalise and adapt the treatment to your individual needs when you are receiving treatment over the telephone | **9** |  | **9** |  | **9** |  |
| 21. Practitioner should develop skills to help you to understand your current difficulties/ what has brought you to treatment and set up activities without any written information/ diagrams/pictures (no visual support) | **8** |  | **9** |  | **9** |  |
| 22. Practitioner should develop skills to work together as a team over the telephone without any written information/diagrams/ pictures (no visual support) | **8** |  | **9** |  | **8** |  |
| 23. Practitioner should develop skills to use the symptom questionnaires in a useful way over the telephone (e.g. using your questionnaire answers to decide the focus of the session) | **8** |  | 6 | 6 | **7** |  |
| 24. Practitioner should develop skills to assess if it is appropriate for you to receive telephone treatment (e.g. learning difficulties, hearing problems) | **9** |  | **9** |  | **9** |  |
| 25. Practitioner should develop skills to explore what you expect from treatment, provide information on the type of treatment you will be receiving (guided-self-help treatment) and address your expectations | **9** |  | **8** |  | **8** |  |
| 26. Practitioner should develop skills to explore your feelings and thoughts about working over the telephone | **8** |  | **8** |  | **9** |  |
| 27. Practitioner should develop skills to help you to talk about your experiences over the telephone | **8** |  | **7** |  | **7** |  |
| 28. Practitioner should develop skills to address if you talk too much or help you to talk more if needed when the intervention is delivered over the telephone | **8** |  | **9** |  | **8** |  |
| 29. Practitioner should develop skills to explore your understanding of the things that you are doing/discussing as part of the treatment and checking how you are doing over time when the intervention is delivered over the telephone | **9** |  | **8** |  | **9** |  |
| 30. Practitioner should develop skills to increase your commitment and motivation to change over the telephone *within* the session | **8** |  | **8** |  | **8** |  |
| 31. Practitioner should develop skills to increase your commitment and motivation to change over the telephone *between* sessions (enhance links between sessions) | **8** |  | **9** |  | **8** |  |
| 32. Practitioner should develop skills to know how to handle the session over the telephone if you have not been able to do the homework/activities as planned | **8** |  | **8** |  | **8** |  |
| 33. Practitioner should develop skills to deal with noises and other potential issues about the place you are in/your surroundings when you are answering the session phone call (supermarket, park) | **8** |  | **8** |  | **8** |  |
| 34. Practitioner should develop skills to cope with telephone work demands (e.g. limited time, number of patients per week) | **9** |  | **8** |  | **8** |  |
| **ATTITUDE AND BELIEFS: This section looks at how important is it for you that your practitioner thinks about their attitudes and beliefs related to deliver treatment over the telephone** |  |  |  |  |  |  |
| 35. How important is it for you that your practitioner thinks about their attitudes and beliefs related to deliver treatment over the telephone? | **8** |  | **7** |  | **9** |  |
| **How important is it to you that your practitioner thinks about…** |  |  |  |  |  |  |
| 36. Practitioners should think about the positives/benefits of telephone treatment (including for the patients, practitioners, service) | **8** |  | **7** |  | **8** |  |
| 37. Practitioners should think about the practical reasons that services deliver assessments and treatments over the telephone compared to the health care guidelines available for its use | **7** |  | **7** |  | **7** |  |
| 38. Practitioners should think about how you feel and what you think of receiving treatment over the telephone and how to help you to improve your commitment to work together over the telephone | **8** |  | **8** |  | **8** |  |
| 39. Practitioners should think about their own feelings and thoughts as practitioners about delivering treatment over the telephone and how to improve their commitment towards this mode of working | **7** |  | **7** |  | **8** |  |
| 40. Practitioners should think about what other mental health professionals think and feel about the delivery of treatment over the telephone and how to improve their views’ towards it | 6 | **8** | **8** |  | 6 | 4 |
| 41. Practitioners should think about challenging their own, their patients’ or other people’s beliefs about treatment delivered over the telephone (e.g. “Treatment delivered over the telephone is a lower and cheaper version of therapy”) | **9** |  | **8** |  | **8** |  |
| 42. Practitioners should think about overcoming their personal dislike of treatment delivered over the telephone through training | **9** |  | **7** |  | **8** |  |
| 43. Practitioners should think about overcoming any personal dislike of treatment delivered over the telephone through doing telephone treatment sessions/practice | **9** |  | 6 | **7** | **8** |  |
| 44. Practitioners should think about discussing audios of telephone treatment sessions during university training to identify what they did well and what could be improved | **8** |  | **7** |  | **8** |  |
| 45. Practitioners should be assessed on telephone specific abilities at university training courses before they become qualified as practitioners | **9** |  | 6 | **7** | **7** |  |
| 46. Practitioners should record telephone treatment sessions with patient agreement to be able to think about the session and identify what went well and what could be done better/improved for the next session | **8** |  | 6 | **7** | **8** |  |
| 47. Practitioners should talk with service managers and colleagues about the expectations of their role and whether these fits with the reality of their day-to-day work | **8** |  | **9** |  | **8** |  |
| 48. Practitioners should think about their role as a coach or therapist | **8** |  | **9** |  | **8** |  |
| 49. Practitioners should think about approaching sessions like a ‘teacher’ vs working together with you from a therapy/therapeutic approach | 5 | 6 | **8** |  | **7** |  |
| 50. Practitioners should think about other people’s perceptions of their role | 5 | **8** | 6 | **8** | 6 | 3 |
| **FEELINGS AND INCENTIVES: This section looks at how important is it for you that your practitioner talks about their feelings related to deliver treatment over the telephone, and how important is for you to help practitioners identify reasons to inspire them to deliver treatment over the telephone** |  |  |  |  |  |  |
| 51. How important is it to you how your practitioner feels about delivering treatment over the telephone? | **8** |  | **7** |  | **8** |  |
| 52. How important is it to you that your practitioner talks to service managers and colleagues about their feelings related to delivering treatment over the telephone (e.g. anxiety, uncertainties)? | **8** |  | 6 | **7** | **9** |  |
| 53. How important is it to you that your practitioner talks to service managers and colleagues about possibly feeling undervalued? | **7** |  | **8** |  | **8** |  |
| 54. How important is it to you that your practitioner is motivated to deliver treatment over the telephone? | **9** |  | **8** |  | **8** |  |
| 55. How important is it to you that your practitioner knows that you had a positive experience of treatment delivered over the telephone to feel motivated to continue delivering treatment in this modality? | **9** |  | **9** |  | **9** |  |
| 56. How important is it to you that your practitioner is able to offer choices to you about how you receive treatment (for example, over the phone or face-to-face) to feel motivated to continue delivering treatment in this modality? | **9** |  | **8** |  | **8** |  |
| **Section B:** We are interested in what you think we should be asking **services** to change/do differently to: (a) improve delivery by practitioners of psychological interventions over the telephone for patients with anxiety and/or depression, (b) improve engagement by patients with psychological interventions delivered over the telephone | **9** |  | **8** |  | **8** |  |
| 57. How important is it to you that NHS services provide specific training to your practitioner about treatment delivered over the telephone before they contact you? | **9** |  | **9** |  | **8** |  |
| **How important is it to you that NHS services provide the following information to your practitioner before they contact you over the telephone:** |  |  |  |  |  |  |
| 58. Information related to sharing materials with you when the intervention is delivered over the telephone (post, email, before/after the session, workbook/worksheet) | **7** |  | **9** |  | **8** |  |
| 59. Information about what to do over the telephone if they are concerned about your welfare/keeping you safe if you are at risk (knowing what do if your practitioner is working at the service and if he/she is working from home) | **9** |  | **9** |  | **9** |  |
| 60. Information on ending sessions/discharge procedures for treatments delivered over the telephone | **9** |  | **8** |  | **8** |  |
| 61. Information on what to do if you do not answer the phone call or you lose phone contact/communication in the middle of a session, e.g. number of times to call you back, leaving a voice mail | **9** |  | **9** |  | **8** |  |
| 62. Information on what to do if you answer the call when you are in a supermarket or a park, places that are not confidential and private | **8** |  | **9** |  | **9** |  |
| 63. Information on what to do if you did not manage to do the homework/activities as planned | **8** |  | **7** |  | **7** |  |
| **PRACTITIONER WORKING ENVIRONMENT: This section looks at what kind of working environment you think NHS services should provide to your practitioner before they start delivering psychological treatment over the telephone.** |  |  |  |  |  |  |
| 64. How important is it to you the environment in which your practitioner delivers treatment over the telephone? | **8** |  | **9** |  | **8** |  |
| **Please consider the importance of each of the following items referring to your practitioner’s working environment:** |  |  |  |  |  |  |
| 65. Practitioners’ working environment should help them to focus their attention on what you are saying over the telephone (e.g. remove distractions) | **7** |  | **9** |  | **9** |  |
| 66. Practitioners should work in small offices shared with 4-6 colleagues when they are talking over the telephone with you | **7** |  | **7** |  | 5 | 5 |
| 67. Practitioners should work in an individual private office when they are talking over the telephone with you | 5 | 5 | **8** |  | 5 | 5 |
| 68. Practitioners should work in a shared open plan office with other practitioners who are delivering telephone treatment | 6 | 5 | 5 | **4** | 3 | 4 |
| 69. Practitioners should work in a shared open plan office with other practitioners that may or may not deliver telephone treatment | 4 | 5 | 4 | **2** | 2 | 3 |
| 70. Practitioners should be allowed to work from home when they are delivering treatment over the telephone | 5 | 5 | **8** |  | 6 | 5 |
| **RESOURCES/EQUIPMENT: This section looks at what resources/equipment you think NHS services should have in place to deliver treatment over the telephone.** |  |  |  |  |  |  |
| 71. How important is it to you that the NHS service counts with the resources/equipment that is needed to deliver treatment over the telephone? | **7** |  | **9** |  | **8** |  |
| **Please consider the importance of each of the following items referring to resources/ equipment to deliver treatment over the telephone:** |  |  |  |  | **9** |  |
| 72. Different options should be available to share materials with you and to meet your needs (e.g. email, post, on-line) | **8** |  | **9** |  | **9** |  |
| 73. A number of headsets and good quality headsets should be available to deliver treatment over the telephone | **9** |  | **9** |  | **8** |  |
| 74. Letting you decide on how you receive psychological treatment (e.g. face-to-face, telephone, group, online) | **9** |  | **9** |  | **8** |  |
| 75. Flexibility to offer you an assessment and/or the first treatment session face-to-face | **8** |  | **8** |  | **8** |  |
| 76. Being able to identify the mode of treatment received by patients (for example, between patients that received treatment face-to-face and those that received telephone treatment) within the electronic records | **8** |  | **7** |  | **8** |  |
| 77. Have equipment available to record telephone sessions | **7** |  | 6 | 6 | **7** |  |
| 78. Reduce practitioner preparation time before delivering a telephone session by having easily accessible materials (e.g. printed worksheets) | **8** |  | **7** |  | **8** |  |
| 79. Increase and acknowledge the time it takes the practitioner to prepare for sessions being delivered over the telephone | **9** |  | **9** |  | **7** |  |
| 80. Flexibility to deliver treatment using different modalities and not mainly telephone, i.e. face-to-face and telephone | **8** |  | **9** |  | **8** |  |
| **SOCIAL OPPORTUNITY: This section looks at what opportunities you think NHS services should offer to your practitioner to ensure they receive the support they need to deliver treatment over the telephone.** |  |  |  |  |  |  |
| 81. How important is it to you that NHS services provide support to your practitioner to deliver treatment over the telephone? | **9** |  | **8** |  | **8** |  |
| **Please consider the importance of each of the following items to facilitate engagement and quality of treatment delivered over the telephone:** |  |  |  |  |  |  |
| 82. Service being able to have arrangements in place to allow practitioners joining the service to observe/shadow an experienced colleague working over the telephone before delivering treatment over the telephone | **8** |  | **9** |  | **8** |  |
| 83. Service providing initial close supervision to assess your practitioner’s telephone skills/abilities and performance on the delivery of treatment over the telephone | **9** |  | 6 | **7** | **8** |  |
| 84. Service providing regular meetings with an experienced senior practitioner (supervision) to help your practitioner to develop telephone skills and increase their confidence | **8** |  | **8** |  | **9** |  |
| 85. Service should ask your practitioner to record telephone treatment sessions with you so then their delivery of telephone treatment could be assessed and discussed privately/confidentially in supervision | **7** |  | 5 | 6 | **7** |  |
| 86. Service having telephone treatment champions (specific members of staff within the service who increase awareness and support for treatment delivered over the telephone) | 6 | **9** | 6 | 4 | 6 | 3 |
| 87. The clinical manager of your practitioner should have experience delivering telephone therapy | **8** |  | **8** |  | **7** |  |
| 88. The clinical manager of your practitioner should have a positive view/attitude towards telephone treatment | **9** |  | **8** |  | **9** |  |
| 89. Your practitioner should receive support to think about reasons that might be affecting/interfering with your recovery over the telephone (i.e. not blame the telephone without having a strong reason/logic for it) | **8** |  | **8** |  | **8** |  |
| 90. Your practitioner should have opportunities for professional development (ongoing training, booster skills sessions) | **7** |  | **9** |  | **9** |  |
| 91. Service should promote working together as a team where practitioners can offer each other peer support and advice about treatment delivered over the telephone | **8** |  | **8** |  | **8** |  |
| **Section C:** We are interested in what you think we should change/do differently at **a community level** to: (a) improve delivery by practitioners of psychological interventions over the telephone for patients with anxiety and/or depression, (b) improve engagement by patients with psychological interventions delivered over the telephone |  |  |  |  |  |  |
| 92. How important is it that your GP is knowledgeable about the psychological treatment they refer patients to? | **8** |  | **9** |  | **9** |  |
| 93. How important is it that the public is aware of the variety of different psychological treatments (e.g. not just counselling) and its different modalities (e.g. not just face-to-face)? | **9** |  | **9** |  | **8** |  |

**Note:** Numbers in bold were rated as “extremely important” (i.e. median => 7). Domains rated below 7 on Round 1 were then discussed and rated as part of Round 2.
