# Supplementary material for: Enhancing the Behaviour Change Wheel with synthesis, stakeholder involvement and decision-making: a case example using the ‘Enhancing the Quality of Psychological Interventions Delivered by Telephone’ (EQUITy) research programme
Source: Implement Sci. 2021 May 14;16:53. doi: 10.1186/s13012-021-01122-2 (PMC8120925; doi:10.1186/s13012-021-01122-2)
Supplement: Supplementary file 11 — Additional file 11. Minutes from moderated discussions from domains rated as “Not important” (i.e. Median <7) at Round 1 [file 13012_2021_1122_MOESM11_ESM.docx]

**Additional File 11.** Minutes from moderated discussions from domains rated as “Not important” (i.e. Median <7) at Round 1

1. ***Minutes from moderated discussions with Patients***

**Item 7:** To know that your practitioner might be typing notes into the computer during the telephone session.

- Helpful to receive an explanation at the beginning of the session that the practitioner may be typing during the telephone session.
- As long as it does not interfere with the therapy “I’m ok with it”.
- One attendee suggested the NHS Service just need to get better keyboards.

**Item 40:** Practitioners should think about what other mental health professionals think and feel about the delivery of treatment over the telephone and how to improve their views towards it.

- What is important to the patient is the practitioner’s ability to deliver telephone therapy.
- “Nice” if they receive support from their colleagues as it will increase practitioner confidence but for the patient it does not matter – what matters for the patient is that the practitioner delivering telephone is on board with telephone therapy despite people around them being dismissive.
- One attendee noted that they did not fully understand the question.

**Item 49:** Practitioners should think about approaching sessions like a ‘teacher’ vs working together with you from a therapy/therapeutic approach.

- Did not like the word ‘teacher’ (a problematic word could trigger bad memories from school environment). Attendees suggested words such as ‘facilitator’, ‘mentor’, and ‘peer’ to describe the practitioner approach.
- Practitioners provide information and techniques that you might not be aware of
- Do not like feeling the practitioner is reading from a script (hear typing) – feel practitioner not taking it seriously.
- Definitely need a therapeutic approach, therapeutic relationship – the practitioner gently raises patient awareness. One attendee noted when reflecting on previous CBT treatment sessions that they were been taught something but it did not feel that way at the time.

**Item 50:** Practitioners should think about other people’s perceptions of their role.

- In any job you would like to be valued but “I care [as a patient] about it if the person delivering telephone values it”.
- The practitioner surroundings in relation to what other colleagues may be thinking about telephone therapy does not bother the patient as long as the practitioner is able to deliver the therapy, have confidence in the therapy and value the therapy.
- If it is a new approach usually people [other colleagues] might not be “pro it”.
- More important practitioner delivering the therapy believes and trusts in it but “nice” if colleagues do too.
- Practitioners definitely need support from people higher up.

**Item 67:** Practitioners should work in an individual private office when they are talking over the telephone with you.

- This is a question for the practitioner.
- “Not bothered about the working environment as long as I receive the care I need”.
- Choices available to the practitioner so they can decide where they take the calls (private office, shared area).

**Item 68:** Practitioners should work in a shared open plan office with other practitioners who are delivering telephone treatment.

- Do not think this bothers us [patients] – it is the extent this effects the practitioner.
- More a question for the practitioner.
- As long as it does not affect the therapy.
- As long as there is not much background noise.

**Item 69:** Practitioners should work in a shared open plan office with other practitioners who may or may not deliver telephone treatment.

Discussed in relation to question 67

**Item 70:** Practitioners should be allowed to work from home when they are delivering treatment over the telephone.

- Concerned about confidentiality – how do you ensure this if the practitioners are working from home - may be other people around.
- Potentially more distractions for the practitioner when working from home.
- Think practitioners should be working from an office not from home (one attendee expressed that they felt strongly about this).
- More experienced people could work from home but less experienced people may need support and therefore suggested they work from an office within the service.
- Some attendees stated that patients might not know where the practitioner is working from when they receive telephone sessions – patient is unaware of this.
- Focus for the patient is quality of care/standard of delivery not the working environment of the practitioner.
- One attendee noted that they were not sure if the session is recorded when practitioners are working from home or from the service.

**Item 86:** Service having telephone treatment ‘Champions’ (specific members of staff within the service to increase awareness and support for treatment delivered over the telephone).

- Criteria to become a ‘champion’ discussed - whether this would be experience/skills or attitude. How many years do they need to become a ‘champion’? Someone with lived experience of mental health difficulties? A professional?
- One attendee suggested observing the ‘champions’ and comparing the ‘champions’ with someone who is not a ‘champion’ to see if they say the right phrases and how this impacts on the final outcome.
- If other services have the ‘champion’ strategy in place – check how this works for them.
- Noted that this question is more relevant for practitioners as it’s “a team thing” - something the patient does not see.
- Staff to comment on this – practitioners need to define what a ‘champion’ is or is not.
- ‘Champion’ is about motivation within the team
- Patient as a ‘champion’

1. ***Minutes from moderated discussions with Practitioners***

**Item 6: How important is it to you that your patient has written information about your experience (a bio) and a picture of you?**

- Unclear about what to include in the bio because practitioners will have different backgrounds – concerns about not presenting well to patients and leading to discrimination about age. If it’s the PWP’s first job and she/he doesn’t have much experience, or looks very young, judgements/discrimination could be made by patients. This could make the PWP feel uncomfortable. It could lead to age discrimination.
- Some patients might like anonymity
- Wouldn’t provide a bio if it were f2f – it therefore seems unnecessary.
- There was a trial where a bio was sent to patients – the feedback was that it was nice to be able to see the PWP – not sure about the impact of this information in therapeutic alliance and outcomes, impact on the session. Do not do it now.

[JC discussed the patient data]

- Patients seemed to be usually confused to what is a PWP and who they are seeing, one attendee was interesting to know how many patients would be able to say correctly who they have seen, they may say they had CBT but this is not correct, they may have received guided-self-help
- Sounds like the patient wants to understand more about what a PWP is. Some patients say they have had CBT and it didn’t work but it turns out it was not CBT and was guided self-help. Clients need to know what they have had and what they have not had so they know what’s available and it’s ‘not the end of their journey’.
- It might make the PWP feel uneasy that patients could identify her/him outside work but she/he wouldn’t know them. Not comfortable with the patient having a photograph of them
- If you see the patient F2F they would normally have a better idea of your role and who are they seeing
- The expertise of the PWP influences the view on bios, pending on level of expertise

[JC spoke]

- The importance of managing expectations and patient assumptions
- Sometimes there’s an assumption regarding therapeutic alliance when the treatment is delivered over the telephone – So it is important to ask the patient at the end of the session?
- CA summarised that we would have to be very careful about our recommendations – patients were keen but were aware of the nuances discussed.

**Item 7: How important is it to you that your patient knows you might be typing notes into the computer during the telephone session?**

- It’s important that patients know that PWPs are typing notes on the computer – they might hear typing in the background and might worry, questioned, may think that they’re not listening or not interested or that we are not paying attention to them. Confidentiality also needs to be explained. Client might clam up if they hear typing.
- PWPs don’t mention taking notes if f2f – stereotyping the view that they are writing something ‘really important’ and it means something and that could interfere in what they say next
- Patient might think what they said must be something important if they hear typing
- I always say to my patients that ‘I ‘m taking notes, as I do with f2f’ , ‘I’ll be typing’
- Can manage silences by saying ‘I’m just making a note of that’
- Some people are good at typing and listening and some are not
- I’ll say I’m typing so the patient is aware and does not think that I am being distractive
- By session 5 I probably not be typing quite so much but if silent I’ll state ‘I’m just making a note of that’

**Item 23: How important is it to you to develop skills to use the symptom questionnaires in an integrative way over the telephone (e.g. using patient questionnaire answers to decide the focus of the session)?**

- It’s important to check the scores – particularly the risk question and to home in on specific scores/things. Patient can communicate through scores and not mention it directly in the session therefore it’s important to integrate into the session.
- Would expect questionnaires to be integrated in the sessions and wonder whether there would be anything different about it on the telephone. If they score high in some particular areas you will be discussing that with the patient within the session, so I wonder what’s different over the telephone

[CA and CF spoke - there’s the view that it’s used as a tick-box exercise]

- Wonder if it’s been used differently to F2F? Or if they are using them as a tick-box exercise F2F too? This item has been considered unimportant by some attendees because people feel they are doing it anyway, exactly the same as f2f
- I scored middle of the range because I thought it’s the same as F2F – the QAs give focus/direct the session
- Questionnaires and what patients are saying are equal components in directing the session

**Item 43: How important is it to you to overcome any personal dislike of treatment delivered over the telephone through practice?**

- CA noted same as Question 42 - the wording of the question has an impact on how you answer it – don’t need to correct your attitude if it is positive, but if your attitude is negative, then it would be ranked very important [CA summed this up for one attendee]

**Item 45: How important is it to you to be assessed on telephone specific abilities at university training before you become qualified?**

- This could have been something that would have been helpful – doing ok without specific training but in hindsight it would have been really helpful/valuable
- Assessed competency tool is the same for telephone as f2f in our training at Manchester
- UCLAN – first assessment is telephone and then F2F
- First assessment is a telephone assessment – it has been since we trained 7 years ago
- Evaluation of treatment is lost in all modalities, there is not much evaluation on the training including treatment, but this is applicable for F2F and Telephone. The assessment during training corresponds to 1 treatment session only.
- Not assessed on the individual interventions for F2F or Telephone
- Important to have a two-day training on this – everything is usually really rushed/feels rushed on the course. Need to invest time in it - if PWPs feel they are being trained properly and there is time given to it, PWPs will invest more and you’ll get an investment from them
- Only taught seven interventions - you only get half a day on each intervention. You just get an overall mark at the end rather than individual assessment marks. At university you do not have an assessment on each of them. If this is a core aspect of our work it Is important to be invested and to be assessed
- Its’ great that you can submit f2f or telephone assessment. If you can’t submit a telephone assessment, it’s sending a message that F2F is more important and that the telephone session is not good enough to submit
- University and service – when qualified you’re anxious about telephone – you’ve not had the training
- Treatment time is important and this is not about being therapeutic or not, is about spending time in what you need in training

**Item 46: How important is it to you to record telephone treatment sessions with patient agreement to reflect on the session to improve performance?**

- It depends which service you work with – we don’t record F2F. I am only recorded on – it once a year. I’m ‘on the fence, wasn’t sure’, think it would be really helpful, but it would depend on how often it’s done, when would we be allowed the time? Think it’s always important to reflect on work and learn. I’ve done 7 years – reflecting – and I still learn
- Service constraints – how often/when?
- It’s always important to reflect and it helps you learn
- It’s helpful just to be able to build on your confidence, reflect on what went well
- It was suggested by an attendee that low scores for this item might reflect a reluctance to be scrutinised (‘know it’s good for us but I do not like to feel scrutinised…’)

**Item 50: How important is it to you to reflect on other people’s perceptions of your role?**

- Didn’t understand the question – who are ‘other people’? *some agreement from other attendees. It was explained that other people included colleagues, other people in the service, patients, GPs, etc
- Bad experience with GP regarding a risk issue recently – it would help if they had a better understanding of what we do. GP should have some information – take our concerns seriously
- In general, there is a poor understanding of the PWP role, we are easily accessible but no one knows who we are and what we do – National IAPT Service, all about psychologists and about CBT. Society-wide no one knows about our role, people assume, GP… Nobody’s talking about IAPT, people don’t know how it works – GPs also made the same assumptions, they think we are counsellors, they do not know what we do
- We are often referred to as ‘counsellors’ by GPs - they are feeding the wrong information to patients which impacts on their expectations and when the PWP is explaining that he/she is not a counsellor the patient is resistant because the GP said he/she needs to see a counsellor
- We feel powerless to address this, and this is not our role. My role does not involve talking to GPs, so I cannot change how they think
- We tried to address it by giving presentations to GPs – often difficult to get to see them but we did many presentations in surgeries with GPs, we explained about IAPT, about the PWP role, the steeped care model, but nothing has changed
- GPs have such negative views to what PWPs do, then we need to unravel the expectations from clients
- PWPs seen as not professional, don’t value the service provided and wouldn’t take referrals from PWPs (only from Nurses and Social Workers)
- PWPs are not given acknowledgement generally in society. GPs don’t feel it is worthy treatment and often undermine recommendations made by PWPs
- PWPs expected to see everyone from early stages
- GPs will undermine what you have offered a client and will say client needs 1:1 counselling if the PWP has offered a group course
- The GP will tell the patient he/she needs F2F, so when we offer group or telephone, the patient says but my GP said I need F2F
- GPs do not understand what we do and I fear that if we offer telephone the GP would undermine it. They’ve said groups are no good to the patient and they need F2F but we have good recovery rates for groups
- There’s high numbers of clients and they don’t care what we do with them sometimes. Clients want F2F but when we ask why they reply ‘don’t know just do’

**Item 52: How important is it to you to discuss with service managers and colleagues your feelings related to delivering treatment over the telephone (e.g. anxiety, uncertainties)?**

- Attendees not anxious (low scores) but would be helpful in a training intervention
- Training will help to benefit people if they are feeling anxious about the delivery of treatment over the telephone [summed up by CA]

**Items 68 – 69:**

**68. How important is it to you to work in a shared open plan office with other practitioners who are delivering telephone treatment?**

**69. How important is it to you to work in a shared open plan office with other practitioners who may or may not deliver telephone treatment?**

- I misread it – how important is it that you’re in that environment vs whether it’s a good space
- Open plan office is helpful (if there is not too much the noise) because you can talk to a colleague immediately re call and share experience – less isolating
- I’m complete opposite – hate working in a large office. Work from home as it’s quiet, I can concentrate. Step 2 managers sometimes have complaints from patients about the background noise – not confidential
- Focus more at home, quiet, can give more to the client
- Sometimes you can be very self-conscious of what you’re saying in the office mainly if there is a very sensitive issue because you would like to get it right, because you are aware that people is listening Reading off a script – all saying same thing, not personal, sounding like a call centre when you all are saying the same thing
- Feel for the client
- Concerns re confidentiality when the person on the phone hears another client
- Suppose to be confidential – PWP may state ‘ I think I know the person they are on the phone to – need to leave the room now’
- PWPs are asking patients to find a quiet space, but PWPs doing the opposite
- There are benefits of telephone working in terms of time – saving on room space
- Work from home – Is a potential solution
- The background noise is a problem and a distraction
- Some services don’t allow working from home – lack of trust.
- Working in an office with 20 people can be frustrating and there is background noise; some PWP’s don’t find other PWPs distracting at all. Some people there are loud and in the office for other reasons and are not delivering telephone treatment. More like a ‘call centre’. Smaller offices good if things are ‘rough’
- F2F – you shut the door – it’s just the two of you
- Like working on my own at home - despite liking the idea of working alone from home, I am lazy and would get distracted and wouldn’t work efficiently.
- For this reason, working some days in the office is helpful.
- I get distracted in the office – prefer home
- It is important to have that balance
- Office – space at the back which is more quiet and it has sound proof– good to have that flexibility and that option when you are delivering telephone sessions
- Staff wellbeing – at home can keep on top of things, go for a run
- Depends on the Service – ‘home’ they might think you’re not doing anything
- Love to work from home but in the first year, I learnt so much from the colleagues around me, from more experienced practitioners and that was really helpful.

[Maybe kind of a progression system? CA – Working in the office at first and then moving to work from home]

- Choice and control – flexibility – home for telephone assessment and have office time for other things. If home all the time could become disconnected with it all

**Item 77: How important is it to you that the NHS service has equipment available to record telephone sessions?**

- Need equipment to be able to do that – otherwise you’re scrambling around in drawers
- Rated as not that important as not sure of the purpose beyond supervision and assessment
- Reflective practice – if bring into Step 2 – useful if we had time to do that but I do not think that will be the case
- Recordings are used for supervision and assessment initially
- Scored low – I have an annual observed practice – the supervisor sat next to me – didn’t need recording equipment and that was a big advantage for me

**Item 83: How important is it to you that the NHS service provides INITIAL close supervision to assess your telephone skills and performance on the delivery of treatment over the telephone?**

- When anyone is newly qualified, close supervision is available and would include telephone work, so this is something that is already there going on
- Would be interested to find out how many supervisors are still doing telephone work
- Supervisors should be skilled over the telephone but they seemed to have all sort of different backgrounds

**Item 85: How important is it to you that the NHS service asks you to record telephone treatment sessions for assessment and supervision purposes?**

- Don’t record f2f so don’t see why would record telephone
- Recordings are used in our service and are brought to supervision and discussed with peers – daunting at first, but helpful and I quite like it now – keeps quality check
- For people qualified a long time ago it would be an opportunity for quality checks
- Bad habits develop over time when I was shadowing a colleague when I first started – Supervision is important as there is an opportunity to address that and ensure quality on delivery
- It depends on the focus of the supervision - IAPT services are in danger of micromanaging and contributing to the burn-out effect

**Item 86: How important is it to you that the NHS service has telephone treatment champions (specific members of staff within the service who increase awareness and support for treatment delivered over the telephone)?**

- Services like to create champions, but don’t always practically happen – nice to put on a CV but does it have an impact on what happens on the ground? What do we achieve with champions?
- CA – is part of the problem the word ‘champions’? Some people have a negative connotation of the word
- A champion could be someone you can talk to, like someone used as a reference for telephone treatment, someone to shadow, someone that may have templates of how to do things that is there in the service. But if this is rolled out it could be done in a more formal way, maybe
- A champion could motivate other people and it could be a good idea but it should be someone with training and experience – People with more motivation to find out things, e.g trainings, but then it might be that you are not allowed to go…
- Some services don’t champion telephone treatment – what’s the point of having a champion?

1. ***Minutes from moderated discussions with Key Informants***

**Item 6: How important is it that patients has written information about practitioner’s experience (a bio) and a picture of them?**

- Might be useful for picture – not normal to have experience list in F2F as you can explain role etc at the beginning of the session.
- Similar regarding bio – it’s doing something additional that you wouldn’t do F2F. Based on previous training I’ve received regarding telephone treatment which highlighted anonymity – a picture for me undermines that, removes this. Feels less useful.
- Equally previous trials where they’ve sent out a picture of practitioners – humanises the voice. (perceived importance in research trials) However, delivering therapy over the telephone for years, I don’t think anyone’s said can you send me a picture when delivering therapy by telephone.
- I’m on the fence – I can see how it would be nice to put a face to the voice
- Low rent thing to do – easy – why wouldn’t we? Just bio
- Don’t think bio is a good idea – IAPT client expectations are a lot higher. People can question this. Pressure on the practitioner to write a bio – it’s not healthy. Level of experience could be problematic.
- Also, we do not use a bio for any of the other healthcare professionals within the NHS, why use this with PWPs? Don’t do it in any other intervention or health care setting – GP/Nursing
- Same – they don’t do this elsewhere in the NHS – If the bio and the picture were separate questions I would have rated the bio (1) and picture (5)
- I gave it a 4 – treated many without it – can’t be that important.
- A lot of drop out in IAPT. How people come in and how it’s received. What are the hurdles and what is there to overcome? A picture of the building may be helpful – less concerned regarding providing a picture of the therapist. How we welcome people – worth considering. Aim of welcome – to support and encourage. There’s a volume of people who don’t get to the treatment part.

[Can you sum up something Therapist could say?]

How we receive people in to a service. Support people in a place where they are vulnerable. To divert from services – maybe structural things we could do.

- Do this earlier – orientation session
- Not telephone specific – a service thing. Photographs on the website – information available.
- Service issue – how IAPT is more welcoming, the first impression really counts (gives an example about your first impression when you go to a hotel). The importance of meeting patient expectations. Good ‘customer service’.
- Wouldn’t do it for just telephone – blanket across treatment modalities.
- End of session, still people don’t know practitioner’s name and title – these things are not always important for others.
- Orientation sessions could be implemented, this is the service welcoming the patients and if implemented could be for every modality of treatment
- How you promote service – engagement is important. Difficulties to consider. If the session is at home they will not have specifics of the person at the end of the phone.

[Research Team – mentioned patient concerns regarding whether practitioner delivering telephone treatment had same level of qualifications as a practitioner delivering F2F]

- Activation letter/leaflet to provide information regarding the type of practitioners, same people, could be pictures of a handful of practitioners from the service on the leaflet. Practical element to how we get that information over. This will be practical because there is a lot of staff turnover, so if the picture is implemented, when someone goes off sick, then the practitioner taking over will need to send a picture, this is not practical.
- Don’t think bio would do that. People may think a post graduate is better than an undergraduate – opening up a can of worms. This will be discriminating between PWPs that are undergrads and those that are not undergrad, but they all received the same training to become qualified. Introduction to treatment rather than a bio would be preferable.
- Study – involved a mixture of F2F and telephone work. This provided a face to the name. If service is pure telephone work – blend the session’s telephone and F2F. You can choose what you put in bio can be to welcome person into treatment.
- Could target client expectations for self-help, remit for Step 2.

**Item 40: How important is it for practitioners to reflect on what other mental health professionals think and feel about the delivery of treatment over the telephone and how to improve their views towards it?**

- This question irritated me – PWPs responsibility to do this or a service responsibility? PWPs are already overworked, stressed, feeling undervalued
- Opportunity to showcase role but this should be a managerial responsibility within the service
- Many PWPs can’t get out of practice to do this, feel they have to justify their work.
- Reflecting on colleagues’ attitudes – not individual key responsibility. Priority should be in structure of the team – service responsibility.
- Educating on the value of the PWP role – not just about giving out leaflets
- Would you want to encourage reflecting as a cohort within the service and the value of their role – depends on who the other mental health professionals are – what value does it add.
- How you share that with clients. PWP unpack with patients why F2F suggested - might be on wonky foundations
- Service priority – question is twofold. Practitioner ability to reflect in supervision. Important their views towards it are service specific.
- Massive issue PWPs undervalued, their role is not clear for others and helping others to recognize the value of PWPs is a service responsibility – getting PWPs to reflect on this could be really depressing for the PWP. Important how other professional views may impact on the patient the PWP is seeing. PWP can’t be addressing other professional views of their role without any support.

**Item 50: How important is it for practitioners to reflect on other people’s perceptions of their role?**

[It has been said this was similar to number 40 and views corresponding to question 40 are applicable to this question]

**Items 66-70: Working environment**

**66. How important is it to work in small offices shared with 4-6 colleagues when delivering treatment over the telephone?**

**67. How important is it to work in an individual private office when delivering treatment over the telephone?**

**68. How important is it to work in a shared open plan office with other practitioners who are delivering telephone treatment?**

**69. How important is it to work in a shared open plan office with other practitioners who may or may not deliver telephone treatment?**

**70. How important is it to be allowed to work from home when delivering treatment over the telephone?**

- Important to be with and share with people who do treatment or not
- Hard to decide – comment on all questions together. Conflicted if working from home is right. Important to be able to work quietly and confidentially with a patient. I worked with district nurses who worked with older patients in a shared environment where the nurses needed to speak loudly over the telephone. Shared environment can address isolation.
- Tele health service – out of hours – PWPs love day at home, working on their own. I’m on the fence – people are different and like different things, that’s why it is important to be flexible and have different options
- Can we let practitioners choose? As long as there is an appropriate risk assessment is done and a confidential space.

[Would this be feasible?]

- Every service needs office space – confidential. Don’t have enough venues, there’s no money for them. Home working is happening already.
- As long as the shared office environment is quiet – not over hearing other people’s phone calls (I was alarmed by recordings submitted to the University Training Course as it didn’t seem to be the case) Is it private? Can people concentrate?
- Can the client hear what’s going on? Staff conversations in the background – times where client has heard. Confidentially breaches in both directions.

[Mix of spaces available appropriate for the task at hand]

- Depends on task – what others are doing e.g. CBT therapist marking etc in the same room but if there’s a risk it’s important to access their help if needed.

[Research team – starting out in shared space and then work from home]

- Training in the office and then work form home
- As long as the shared offices are suitable and provide a confidential quiet environment that is OK, but this is not always the case

[How much time to allow for this?]

- Gradually to then work from home.
- Depends on the treatment. Intervention work should be more straight forward from home.
- Structural things around IAPT – increasing capacity in buildings as a long term plan – IAPT grows but there’s nowhere to go. Push for therapist to move out of the offices. Some GPs charge for rooms and some don’t – getting PWPs back in the community. Regionally and locally – how these aspirations are translated – this will be an issue.
- CPNs not just Step 2 – HIT etc.
- In January we’ll be looking at moving people out – issues regarding limited car park spaces at the office base (i.e. we have 30 people as part of the staff and 10 parking spaces)

**Item 86: How important is it that NHS services have telephone treatment champions (specific members of staff within the service who increase awareness and support for treatment delivered over the telephone)?**

- On the fence – I predominantly think that telephone working is very important – however I’m aware that proponents of this can be irritating for people against telephone treatment
- I agree – idea of ‘champions’ can have the opposite effect – doesn’t work
- Not just champions responsibility – it’s everyone’s. Diminishes responsibility of everyone else.
- Would need to be across the board – including ‘champions’ for F2F. I do not think it will be a good message if you have a champion for telephone because it is giving special treatment to it and it does not sound right, this should be integrated into the service
- Lowers telephone work – telephone work needs to be viewed as business as usual. ‘Champions’ reinforces its different, less valued etc. Needs embedding rather than singling it out.
- Doesn’t have to be a PWP who is the ‘champion’ – help to move it forward.
- I think is about creating a cultural shift within the service, and managerial staff is responsible to do this
- Scored it highly – Leads for everything within the management team spreads over area thinly and this helps to solve problems when the manager is not available, preventing us to receive a lots of emails about the same issue. If there is someone that you can refer too, that could be helpful. Bring problems to the CBT Lead – one person gets an issue e.g. headsets. Helps develop operational skills not just therapy skills.
- Like someone managing the IT side of it.
