# Supplementary material for: Enhancing the Behaviour Change Wheel with synthesis, stakeholder involvement and decision-making: a case example using the ‘Enhancing the Quality of Psychological Interventions Delivered by Telephone’ (EQUITy) research programme
Source: Implement Sci. 2021 May 14;16:53. doi: 10.1186/s13012-021-01122-2 (PMC8120925; doi:10.1186/s13012-021-01122-2)
Supplement: Supplementary file 12 — Additional file 12 a Domains rated at Round 3 as “Essential” (i.e. median between 7 and 9) by patients, practitioners or key informants. b Domains rated at Round 3 as “Not essential” (i.e. median <7) by patients, practitioners or key informants [file 13012_2021_1122_MOESM12_ESM.zip › Additional file 12/Additional File 12aR1.docx]

**Additional File 12a.** Domains rated at Round 3 as “Essential” (i.e. median between 7 and 9) by patients, practitioners or key informants

|  | **Patients (N=7)** | **Practitioners**  **(N=19)** | **Key Informants**  **(N=15)** |
| --- | --- | --- | --- |
| 1. How essential is it that patients know about the psychological treatment that he/she could be receiving over the telephone? | **9** | **9** | **8** |
| 2. How essential is it that patients know telephone appointments are as formal and important as face-to-face? | **9** | **9** | **9** |
| 3. How essential is it that patients know the missed appointment/discharge rules for treatment delivered over the telephone are the same as for face-to-face treatment? | **9** | **9** | **8** |
| 4. How essential is it that patients know treatment over the telephone is being delivered by the same qualified practitioners who deliver face-to-face treatments? | **7** | **9** | **8** |
| 5. How essential is it that patients know he/she should answer the session phone calls in a private, quiet and confidential place? | **8** | **9** | **8** |
| 7. How essential is it that patients know practitioners might be typing notes into the computer during the telephone session? |  | **8** | **7** |
| 8. How essential is it for practitioners to have an overall knowledge about the delivery of psychological treatment over the telephone? | **9** | **8** | **8** |
| 9. How essential is it for practitioners to know about patient experiences of receiving treatment over the telephone? | **9** | **7** | **8** |
| 10. How essential is it for practitioners to know about other practitioner experiences of delivering treatment over the telephone? | **8** |  | **7** |
| 11. How essential is it for practitioners to know about the clinical effectiveness of treatment delivered over the telephone in comparison to face-to-face? | **9** | **8** | **9** |
| 12. How essential is it for practitioners to have 'specific' guidelines to deliver telephone treatment? | **9** | **8** | **7** |
| 13. How essential is it to develop/adapt practitioners’ skill-set to deliver treatment over the telephone? | **9** | **8** | **9** |
| 14. How essential is it to develop/adapt practitioners’ verbal communication skills to suit treatment delivered over the telephone (e.g. verbal empathy)? | **9** | **9** | **9** |
| 15. How essential is it for practitioners to develop skills to adjust their tone of voice over the telephone? | **7** | **8** | **8** |
| 16. How essential is it for practitioners to develop skills to use and deal with silences when talking over the telephone and to recognise signals to know when to talk and when not to talk? | **8** | **8** | **8** |
| 17. How essential is it for practitioners to develop skills to create a feeling/sense of a ‘therapeutic space’ and a ‘safe space to talk’ when delivering treatment over the telephone? | **9** | **9** | **8** |
| 18. How essential is it for practitioners to develop skills to build up a good relationship with patients over the telephone? | **8** | **9** | **9** |
| 19. How essential is it for practitioners to convey active listening when they are delivering treatment over the telephone? | **9** | **9** | **8** |
| 20. How essential is it for practitioners to develop skills to personalise and adapt the treatment to patient's individual need when delivering treatment over the telephone? | **9** | **9** | **8** |
| 21. How essential is it for practitioners to develop skills to help the patient understand the formulation of their current difficulties and set up homework without visual aids (e.g. diagrams)? | **8** | **8** | **8** |
| 22. How essential is it for practitioners to develop skills to work collaboratively with patients over the telephone without visual aids (e.g. diagrams)? | **8** | **9** | **8** |
| 23. How essential is it for practitioners to develop skills to use the symptom questionnaires in an integrative way when they are working over the telephone (e.g. using patient questionnaire answers to decide the focus of the session)? | **8** |  | **7** |
| 24. How essential is it for practitioners to develop skills to assess patient suitability for telephone treatment (e.g. learning difficulties, hearing problems)? | **9** | **9** | **9** |
| 25. How essential is it for practitioners to develop skills to explore, address and manage patient expectations regarding treatment delivered over the telephone? | **9** | **9** | **8** |
| 26. How essential is it for practitioners to develop skills to explore patient feelings and thoughts about working over the telephone? | **9** | **7** |  |
| 27. How essential is it for practitioners to develop skills to help patients to reflect/express their experiences of working over the telephone? | **9** | **7** | **7** |
| 28. How essential is it for practitioners to develop skills to contain more talkative patients and manage less talkative patients when the intervention is delivered over the telephone? | **8** | **9** | **8** |
| 29. How essential is it for practitioners to develop skills to gauge patient understanding and monitor patient progress when the intervention is delivered over the telephone? | **9** | **9** | **8** |
| 30. How essential is it for practitioners to develop skills to increase patient commitment and motivation to change over the telephone WITHIN the session? | **8** | **9** | **7** |
| 31. How essential is it for practitioners to develop skills to increase patient commitment and motivation to change over the telephone BETWEEN sessions (enhance links between sessions)? | **8** | **9** | **8** |
| 32. How essential is it for practitioners to develop skills to manage homework non-compliance over the telephone? | **8** | **8** |  |
| 33. How essential is it for practitioners to develop skills to deal with noises and other potential issues related to the patient's surroundings when answering the session phone call (e.g. supermarket, park, baby crying)? | **8** | **8** | **8** |
| 34. How essential is it for practitioners to develop skills to cope with telephone work demands (e.g. time constraints, number of clinical cases)? | **9** | **8** | **7** |
| 35. How essential is it for practitioners to reflect on their attitudes and beliefs related to delivering treatment over the telephone? | **9** | **7** | **8** |
| 36. How essential is it for practitioners to reflect on the benefits of telephone treatment (including for the patients, practitioners, service)? | **9** | **8** | **8** |
| 37. How essential is it for practitioners to reflect on the practical reasons services deliver assessments and treatments over the telephone compared to the health care guidelines/evidence-base available for its use? | **8** | **7** |  |
| 38. How essential is it for practitioners to reflect on patient attitudes, views and preconceptions of telephone treatment and how to improve patient commitment to working together over the telephone? | **8** | **8** | **8** |
| 39. How essential is it for practitioners to reflect on their attitude, views and preconceptions of telephone treatment and how to improve their commitment towards this mode of working? | **9** | **8** | **7** |
| 40. How essential is it for practitioners to reflect on what other mental health professionals think and feel about the delivery of treatment over the telephone and how to improve their views towards it? | **7** | **9** |  |
| 41. How essential is it for practitioners to challenge their own, their patients’ or other people’s beliefs about treatment delivered over the telephone (e.g. ‘Treatment delivered over the telephone is a lower and cheaper version of therapy’)? | **9** | **7** | **7** |
| 42. How essential is it for practitioners to overcome personal dislike of treatment delivered over the telephone through training? | **9** | **7** |  |
| 43. How essential is it for practitioners to overcome any personal dislike of treatment delivered over the telephone through practice? | **9** | **7** |  |
| 44. How essential is it for practitioners to discuss audios of telephone treatment sessions during university training to assess and improve your performance? | **9** | **8** | **7** |
| 45. How essential is it for practitioners to be assessed on telephone specific abilities at university training before they become qualified? | **9** | **7** | **7** |
| 46. How essential is it for practitioners to record telephone treatment sessions with patient agreement to reflect on the session to improve performance? | **9** | **7** | **7** |
| 47. How essential is it for practitioners to discuss their professional role expectations with service managers and colleagues, and whether these fit with the reality of their day-to-day work? | **9** | **9** |  |
| 48. How essential is it for practitioners to reflect on their role as a coach or as a therapist? | **8** | **9** |  |
| 49. How important is it for practitioners to reflect on approaching sessions like a ‘teacher’ vs working collaboratively with patients from a therapy/therapeutic approach? |  | **8** |  |
| 50. How important is it for practitioners to reflect on other people’s perceptions of their role? |  | **9** |  |
| 51. How essential is it for practitioners to reflect on their feelings regarding delivering treatment over the telephone? | **8** | **8** | **7** |
| 52. How important is it for practitioners to discuss with service managers and colleagues your feelings related to delivering treatment over the telephone (e.g. anxiety, uncertainties)? | **9** |  |  |
| 53. How essential is it for practitioners to discuss with service managers and colleagues about possibly feeling undervalued? | **9** | **9** |  |
| 54. How essential is it for practitioners to be motivated to deliver treatment over the telephone? | **9** | **8** | **7** |
| 55. How essential is it for practitioners to know that patients had a positive experience of telephone treatment to increase their motivation to continue delivering treatment over the telephone? | **9** | **8** | **7** |
| 56. How essential is it for practitioners to feel motivated to deliver treatment over the phone, by having the ability to offer choices to patients about how they want to receive treatment (e.g., telephone, face-to-face, group)? | **9** | **8** | **7** |
| 57. How essential is it that NHS services provide practitioners with 'specific' training on treatment delivered over the telephone before they start using this mode of delivery? | **9** | **8** | **7** |
| 58. How essential is it that NHS services provide practitioners with information related to sharing materials with patients when the intervention is delivered over the telephone (post, email, before/after the session, workbook/worksheet)? | **8** | **8** | **7** |
| 59. How essential is it that NHS services provide practitioners with information about how to proceed over the telephone if they are concerned about patient welfare/safety if your patient is at risk (procedures in place if practitioners are working at the service and if they are working from home)? | **9** | **9** | **9** |
| 60. How essential is it that NHS services provide practitioners with information about discharge procedures for treatments delivered over the telephone? | **9** | **9** | **7** |
| 61. How essential is it that NHS services provide practitioners with information about how to proceed if the patient does not answer the phone call or in case phone contact/communication is lost in the middle of a session (e.g. number of times to call back, leaving a voice mail)? | **9** | **9** | **7** |
| 62. How essential is it that NHS services provide practitioners with information about how to proceed if the patient answers the call from a supermarket or a park, places that are not confidential and private? | **8** | **9** | **7** |
| 63. How essential is it that NHS services provide practitioners with information about how to proceed with homework non-compliance? | **8** | **8** |  |
| 64. How essential is the working environment in which practitioners deliver treatment over the telephone? | **9** | **9** | **7** |
| 65. How essential is it that the working environment facilitates active listening (e.g. remove distractions)? | **9** | **8** |  |
| 66. How important is it to work in small offices shared with 4-6 colleagues when delivering treatment over the telephone? | **7** |  |  |
| 67. How important is it to work in an individual private office when delivering treatment over the telephone? |  | **7** |  |
| 70. How important is it to be allowed to work from home when delivering treatment over the telephone? |  | **7** |  |
| 71. How essential is it that NHS services count with the resources/equipment that are needed to deliver treatment over the telephone? | **7** | **9** |  |
| 72. How essential is it that NHS services have different options available to share materials with patients to meet their needs (e.g. email, post, on-line)? | **8** | **9** | **7** |
| 73. How essential is it that a number of headsets and good quality headsets are available within the NHS services to deliver treatment over the telephone? | **9** | **9** |  |
| 74. How essential is it that NHS services offer options to patients so they can decide on how they would like to receive psychological treatment (e.g. face-to-face, telephone, group, online)? | **8** | **9** |  |
| 75. How essential is it that NHS services provide flexibility to offer patients an assessment and/or the first treatment session face-to-face? | **9** | **7** |  |
| 76. How essential is it that NHS services are able to identify the mode of treatment delivery (e.g., face-to-face, telephone) within the electronic databases? | **8** | **7** |  |
| 77. How important is it that NHS services have equipment available to record telephone sessions? | **8** |  |  |
| 78. How important is it that NHS services reduce practitioners preparation time before delivering a telephone session by having easily accessible materials (e.g. printed worksheets)? |  | **8** |  |
| 79. How essential is it that NHS services increase and acknowledge the time it takes practitioners to prepare for sessions being delivered over the telephone? | **9** | **9** |  |
| 80. How essential is it that NHS service provide flexibility to deliver treatment using different modalities and not mainly telephone, i.e. face-to-face and telephone? | **8** | **9** |  |
| 81. How essential is it that NHS services provide support to deliver treatment over the telephone? | **9** | **9** | **7** |
| 82. How essential is it that NHS services are able to have arrangements in place to allow practitioners joining the service to observe/shadow an experienced colleague working over the telephone before delivering treatment over the telephone? | **9** | **9** | **7** |
| 83. How essential is it that NHS services provide INITIAL close supervision to assess your telephone skills and performance on the delivery of treatment over the telephone? | **9** | **7** | **7** |
| 84. How essential is it that NHS services provide regular supervision to support practitioners on the development of telephone skills and increasing their confidence? | **9** | **7** | **7** |
| 85. How important is it that NHS services ask practitioners to record telephone treatment sessions for assessment and supervision purposes? | **7** |  |  |
| 87. How essential is it that clinical managers have experience delivering treatment over the telephone? | **9** | **8** |  |
| 88. How essential is it that clinical managers have a positive view/attitude towards treatment delivered over the telephone? | **9** | **9** | **7** |
| 89. How essential is it for practitioners to receive support to reflect on reasons that might be affecting/interfering with patient recovery over the telephone (e.g. not blame the telephone without having a strong reason/logic for it)? | **8** | **9** | **7** |
| 90. How essential is it for practitioners to have opportunities with NHS services for professional development (ongoing training, booster skills sessions)? | **8** | **9** | **7** |
| 91. How essential is it for practitioners that NHS services promote working together as a team and facilitate peer support and advice about treatment delivered over the telephone? | **9** | **9** |  |
| 92. How essential is it that GPs are knowledgeable about the IAPT psychological treatments they refer patients to? | **9** | **9** |  |
| 93. How essential is it that the public is aware of the variety of different psychological treatments (e.g. not just counselling) and different methods/modes of delivery (e.g. not just face-to-face)? | **8** | **9** |  |

**Note:** Domains without a number were rated as “not essential” (i.e. median <7)
