# Supplementary material for: Enhancing the Behaviour Change Wheel with synthesis, stakeholder involvement and decision-making: a case example using the ‘Enhancing the Quality of Psychological Interventions Delivered by Telephone’ (EQUITy) research programme
Source: Implement Sci. 2021 May 14;16:53. doi: 10.1186/s13012-021-01122-2 (PMC8120925; doi:10.1186/s13012-021-01122-2)
Supplement: Supplementary file 12 — Additional file 12 a Domains rated at Round 3 as “Essential” (i.e. median between 7 and 9) by patients, practitioners or key informants. b Domains rated at Round 3 as “Not essential” (i.e. median <7) by patients, practitioners or key informants [file 13012_2021_1122_MOESM12_ESM.zip › Additional file 12/Additional File 12bR1.docx]

**Additional File 12b.** Domains rated at Round 3 as “Not essential” (i.e. median <7) by patients, practitioners or key informants.

|  | **Patients (N=7)** | **Practitioners**  **(N=19)** | **Key Informants**  **(N=15)** |
| --- | --- | --- | --- |
| 6. How important is it that patients has written information about practitioner’s experience (a bio) and a picture of them? | **6** | **1** | **1** |
| 7. How important is it that patients know practitioners might be typing notes into the computer during the telephone session? | **4** |  |  |
| 10. How important is it for practitioners to know about other practitioner experiences of delivering treatment over the telephone? |  | **6** |  |
| 23. How important is it for practitioners to develop skills to use the symptom questionnaires in an integrative way when they are working over the telephone (e.g. using patient questionnaire answers to decide the focus of the session)? |  | **6** |  |
| 26. How important is it for practitioners to develop skills to explore patient feelings and thoughts about working over the telephone? |  |  | **6** |
| 32. How important is it for practitioners to develop skills to manage homework non-compliance over the telephone? |  |  | **6** |
| 37. How important is it for practitioners to reflect on the practical reasons services deliver assessments and treatments over the telephone compared to the health care guidelines/evidence-base available for its use? |  |  | **6** |
| 40. How important is it for practitioners to reflect on what other mental health professionals think and feel about the delivery of treatment over the telephone and how to improve their views towards it? |  |  | **3** |
| 42. How important is it for practitioners to overcome personal dislike of treatment delivered over the telephone through training? |  |  | **6** |
| 43. How important is it for practitioners to overcome any personal dislike of treatment delivered over the telephone through practice? |  |  | **5** |
| 47. How important is it for practitioners to discuss their professional role expectations with service managers and colleagues, and whether these fit with the reality of their day-to-day work? |  |  | **5** |
| 48. How important is it for practitioners to reflect on their role as a coach or as a therapist? |  |  | **6** |
| 49. How important is it for practitioners to reflect on approaching sessions like a ‘teacher’ vs working collaboratively with patients from a therapy/therapeutic approach? | **4** |  | **5** |
| 50. How important is it for practitioners to reflect on other people’s perceptions of their role? | **5** |  | **3** |
| 52. How important is it for practitioners to discuss with service managers and colleagues your feelings related to delivering treatment over the telephone (e.g. anxiety, uncertainties)? |  | **6** | **6** |
| 53. How important is it for practitioners to discuss with service managers and colleagues about possibly feeling undervalued? |  |  | **4** |
| 63. How important is it that NHS services provide practitioners with information about how to proceed with homework non-compliance? |  |  | **6** |
| 65. How important is it that the working environment facilitates active listening (e.g. remove distractions)? |  |  | **5** |
| 66. How important is it to work in small offices shared with 4-6 colleagues when delivering treatment over the telephone? |  | **6** | **3** |
| 67. How important is it to work in an individual private office when delivering treatment over the telephone? | **5** |  | **3** |
| 68. How important is it to work in a shared open plan office with other practitioners who are delivering telephone treatment? | **4** | **4** | **3** |
| 69. How important is it to work in a shared open plan office with other practitioners who may or may not deliver telephone treatment? | **4** | **2** | **2** |
| 70. How important is it to be allowed to work from home when delivering treatment over the telephone? | **5** |  | **3** |
| 71. How important is it that NHS services count with the resources/equipment that are needed to deliver treatment over the telephone? |  |  | **4** |
| 73. How important is it that a number of headsets and good quality headsets are available within the NHS services to deliver treatment over the telephone? |  |  | **4** |
| 74. How important is it that NHS services offer options to patients so they can decide on how they would like to receive psychological treatment (e.g. face-to-face, telephone, group, online)? |  |  | **6** |
| 75. How important is it that NHS services provide flexibility to offer patients an assessment and/or the first treatment session face-to-face? |  |  | **4** |
| 76. How important is it that NHS services are able to identify the mode of treatment delivery (e.g., face-to-face, telephone) within the electronic databases? |  |  | **3** |
| 77. How important is it that NHS services have equipment available to record telephone sessions? |  | **6** | **6** |
| 78. How important is it that NHS services reduce practitioners preparation time before delivering a telephone session by having easily accessible materials (e.g. printed worksheets)? | **6** |  | **5** |
| 79. How important is it that NHS services increase and acknowledge the time it takes practitioners to prepare for sessions being delivered over the telephone? |  |  | **5** |
| 80. How important is it that NHS service provide flexibility to deliver treatment using different modalities and not mainly telephone, i.e. face-to-face and telephone? |  |  | **5** |
| 85. How important is it that NHS services ask practitioners to record telephone treatment sessions for assessment and supervision purposes? |  | **5** | **6** |
| 86. How important is it that NHS services have telephone treatment champions (specific members of staff within the service who increase awareness and support for treatment delivered over the telephone)? | **6** | **4** | **2** |
| 87. How important is it that clinical managers have experience delivering treatment over the telephone? |  |  | **2** |
| 91. How important is it for practitioners that NHS services promote working together as a team and facilitate peer support and advice about treatment delivered over the telephone? |  |  | **6** |
| 92. How important is it that GPs are knowledgeable about the IAPT psychological treatments they refer patients to? |  |  | **4** |
| 93. How important is it that the public is aware of the variety of different psychological treatments (e.g. not just counselling) and different methods/modes of delivery (e.g. not just face-to-face)? |  |  | **3** |
