# Supplementary material for: Enhancing the Behaviour Change Wheel with synthesis, stakeholder involvement and decision-making: a case example using the ‘Enhancing the Quality of Psychological Interventions Delivered by Telephone’ (EQUITy) research programme
Source: Implement Sci. 2021 May 14;16:53. doi: 10.1186/s13012-021-01122-2 (PMC8120925; doi:10.1186/s13012-021-01122-2)
Supplement: Supplementary file 13 — Additional file 13. Post-Meeting Experiences for each of the three stakeholder groups [file 13012_2021_1122_MOESM13_ESM.docx]

**Additional File13.** Post-Meeting Experiences for each of the three stakeholder groups

| **FIRST ROUND RATINGS (individual ratings)** | ***Patients***  **(N=7)** | ***Practitioners***  **(N=19)** | ***Key Informants***  **(N=12*)** |
| --- | --- | --- | --- |
| **How easy did you find the task?** | **Frequencies (%)** | **Frequencies (%)** | **Frequencies (%)** |
| Not at all | 1 (14.3) | 1 (5.3) |  |
| A little | 1 (14.3) | 1 (5.3) |  |
| Somewhat | 2 (28.6) | 6 (31.6) | 7 (58.3) |
| Very much so | 3 (42.9) | 11 (57.9) | 5 (41.7) |
| **How difficult did you find the task?** |  |  |  |
| Not at all | 4 (57.1) | 13 (68.4) | 6 (50.0) |
| A little | 2 (28.6) | 5 (26.3) | 4 (33.3) |
| Somewhat |  | 1 (5.3) | 2 (16.7) |
| Very much so | 1 (14.3) |  |  |
| **How clear were the instructions?** |  |  |  |
| Not at all |  | 1 (5.3) |  |
| A little | 1 (14.3) | 1 (5.3) |  |
| Somewhat | 1 (14.3) | 5 (26.3) | 4 (33.3) |
| Very much so | 5 (71.4) | 12 (63.2) | 8 (66.7) |
| **How inconsistent do you believe you were? (due to effects of fatigue, memory, format of instrument, etc)** |  |  |  |
| Not at all | 2 (28.6) | 5 (26.3) | 2 (16.7) |
| A little | 3 (42.9) | 9 (47.4) | 7 (58.3) |
| Somewhat | 2 (28.6) | 5 (26.3) | 3 (25.0) |
| Very much so |  |  |  |
| **SECOND ROUND RATINGS (group ratings)** |  |  |  |
| **How effective did you think the group discussion was?** |  |  |  |
| Not at all |  |  |  |
| A little | 1 (14.3) | 1 (5.3) |  |
| Somewhat | 2 (28.6) | 9 (47.4) | 3 (25.0) |
| Very much so | 4 (57.1) | 9 (47.4) | 9 (75.0) |
| **How informative was the discussion?** |  |  |  |
| Not at all | 1 (14.3) |  |  |
| A little |  | 1 (5.3) |  |
| Somewhat | 1 (14.3) | 12 (63.2) | 1 (8.3) |
| Very much so | 5 (71.4) | 6 (31.6) | 11 (91.7) |
| **How argumentative was the discussion?** |  |  |  |
| Not at all | 2 (28.6) | 14 (73.7) | 8 (66.7) |
| A little | 3 (42.9) | 4 (21.1) | 4 (33.3) |
| Somewhat | 1 (14.3) |  |  |
| Very much so | 1 (14.3) | 1 (5.3) |  |
| **How much did the feedback from the first round ratings influence your second round ratings?** |  |  |  |
| Not at all |  |  | 1 (8.3) |
| A little | 4 (57.1) | 10 (52.6) | 4 (33.3) |
| Somewhat | 3 (42.9) | 7 (36.8) | 7 (58.3) |
| Very much so |  | 2 (10.5) |  |
| **How much did the discussion influence your second round ratings?** |  |  |  |
| Not at all |  |  |  |
| A little | 4 (57.1) | 11 (57.9) | 4 (33.3) |
| Somewhat | 3 (42.9) | 7 (36.8) | 8 (66.7) |
| Very much so |  | 1 (5.3) |  |
| **OVERALL IMPRESSIONS OF YOUR EXPERIENCE** |  |  |  |
| **How much do you think your own ratings will influence the final intervention?** |  |  |  |
| Not at all |  |  |  |
| A little | 3 (42.9) | 7 (36.8) | 6 (50.0) |
| Somewhat | 3 (42.9) | 12 (63.2) | 6 (50.0) |
| Very much so | 1 (14.3) |  |  |
| **How much do you think the group ratings will influence the final intervention?** |  |  |  |
| Not at all |  |  |  |
| A little | 1 (14.3) | 4 (21.1) | 3 (25.0) |
| Somewhat | 2 (28.6) | 6 (31.6) | 4 (33.3) |
| Very much so | 4 (57.1) | 9 (47.4) | 5 (41.7) |
| **How much do you believe that this process can lead to a set of recommendations to improve the quality of therapy delivered over the telephone?** |  |  |  |
| Not at all |  |  |  |
| A little |  | 1 (5.3) | 1 (8.3) |
| Somewhat | 2 (28.6) | 9 (47.4) | 5 (41.7) |
| Very much so | 5 (71.4) | 9 (47.4) | 6 (50.0) |
| **How much do you believe that this process can lead to a set of recommendations to improve patient engagement with therapy delivered over the telephone?** |  |  |  |
| Not at all |  |  |  |
| A little | 1 (14.3) | 1 (5.3) | 1 (8.3) |
| Somewhat | 4 (57.1) | 12 (63.2) | 6 (50.0) |
| Very much so | 2 (28.6) | 6 (31.6) | 5 (41.7) |
| **How satisfying did you find your participation on this meeting?** |  |  |  |
| Not at all |  |  |  |
| A little | 1 (14.3) | 2 (10.5) |  |
| Somewhat | 1 (14.3) | 12 (63.2) | 4 (33.3) |
| Very much so | 5 (71.4) | 6 (31.6) | 8 (66.7) |
| **How did your participation on this meeting compare with your expectations?** |  |  |  |
| Much worse |  |  |  |
| Worse |  |  |  |
| On a par | 2 (28.6) | 6 (31.6) | 8 (66.7) |
| Better | 2 (28.6) | 9 (47.4) | 4 (33.3) |
| Much better | 3 (42.9) | 4 (21.1) |  |

**Note:** *****Data from three key informants were missing.
