# Supplementary material for: Enhancing the Behaviour Change Wheel with synthesis, stakeholder involvement and decision-making: a case example using the ‘Enhancing the Quality of Psychological Interventions Delivered by Telephone’ (EQUITy) research programme
Source: Implement Sci. 2021 May 14;16:53. doi: 10.1186/s13012-021-01122-2 (PMC8120925; doi:10.1186/s13012-021-01122-2)
Supplement: Supplementary file 14 — Additional file 14. Decision-Making: Description of attendees and procedures [file 13012_2021_1122_MOESM14_ESM.docx]

**Additional File 14.** Decision-Making: Description of attendees and procedures

| ***Attendees*** |
| --- |
| The principal investigators (n=2), workstream leads (n=5), the patient and public involvement lead (n=1), the programme manager (n=1) and the researcher associated with the intervention development phase of the programme (n=1) were invited to take part in the meeting. Six of the 10 invited members attended the meeting, which had experience in intervention development, implementation and behaviour change techniques. |
| ***Procedure*** |
| **Before the meeting**  Three days prior to the meeting, data derived from stakeholder meetings (i.e. results from Round 1, Round 2 and Round 3) and minutes from conversations held with each group were circulated (**Additional Files10, 11, 12a & 12b**).  **At the meeting**  The decision-making day meeting comprised two tasks:  1) Results from Round 3 across the three stakeholder groups were presented to identify domains of agreement. For the present purposes we defined agreement as two or more stakeholder groups rating domains as ‘essential’ (i.e. Median >= 7).  2) Intervention functions were identified^[[1]](#footnote-1)^ and, using the Behaviour Change Technique Taxonomy (version 1)^[[2]](#footnote-2)^, behaviour change techniques were proposed for the domains that were identified in the first task. The proposed intervention functions and proposed behaviour change techniques were chosen using the criteria of acceptability, practicability, effectiveness/cost-effectiveness, affordability, safety/side-effects and equity (‘APEASE’)^1^.  **After the meeting**  A document describing the domains proposed for included in the behaviour change intervention, intervention functions for each domain, alongside behaviour change techniques Taxonomy (version 1) and the target level (i.e. patients, practitioners, services, community) were circulated to all the members of the programme team for feedback. On approval, details of the behaviour change intervention were described using the Template for Intervention Description and Replication (TIDieR)^[[3]](#footnote-3)^ (**Additional File 17**). |

1. Michie S, Atkins L, West R. The Behaviour Change Wheel: A guide to designing interventions. Great Britain: Silverback Publishing; 2014. [↑](#footnote-ref-1)
2. Michie S, Richardson M, Johnston M, Abraham C, Francis J, Hardeman W, Eccles MP, Cane J, Wood CE. The behavior change technique taxonomy (v1) of 93 hierarchically clustered techniques: building an international consensus for the reporting of behavior change interventions. Ann Behav Med. 2013; 46(1):81-95 [↑](#footnote-ref-2)
3. Hoffmann TC, Glasziou PP, Boutron I, Milne R, Perera R, Moher D, et al. Better reporting of interventions: Template for intervention description and replication (TIDieR) checklist and guide. BMJ. 2014;348. g1687 [↑](#footnote-ref-3)
