# Supplementary material for: Enhancing the Behaviour Change Wheel with synthesis, stakeholder involvement and decision-making: a case example using the ‘Enhancing the Quality of Psychological Interventions Delivered by Telephone’ (EQUITy) research programme
Source: Implement Sci. 2021 May 14;16:53. doi: 10.1186/s13012-021-01122-2 (PMC8120925; doi:10.1186/s13012-021-01122-2)
Supplement: Supplementary file 15 — Additional file 15. Recommendations: Target levels for the behaviour change intervention [file 13012_2021_1122_MOESM15_ESM.docx]

**Figure 3.** Recommendations: Target levels for the behaviour change intervention


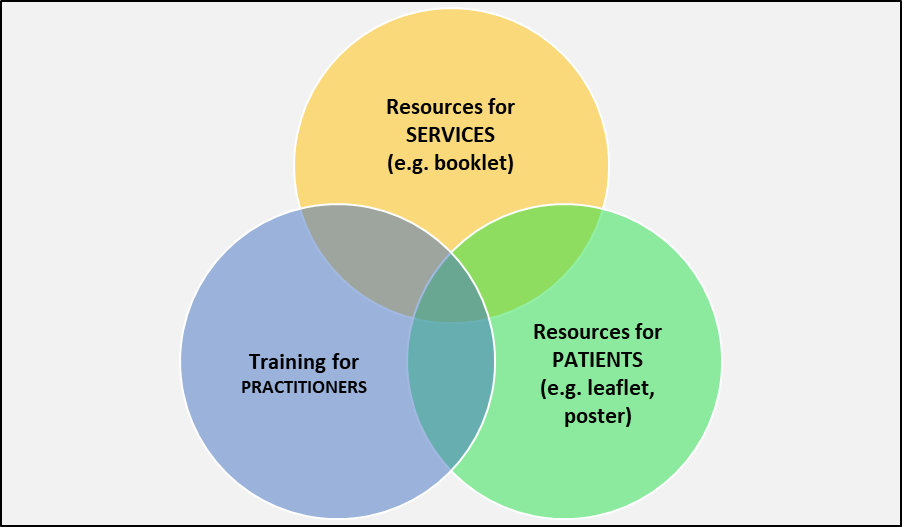


Resources for COMMUNITY

(e.g. leaflet, poster)

As shown in **Figure 2**, the behaviour change intervention has three target levels: services,
