# Supplementary material for: Enhancing the Behaviour Change Wheel with synthesis, stakeholder involvement and decision-making: a case example using the ‘Enhancing the Quality of Psychological Interventions Delivered by Telephone’ (EQUITy) research programme
Source: Implement Sci. 2021 May 14;16:53. doi: 10.1186/s13012-021-01122-2 (PMC8120925; doi:10.1186/s13012-021-01122-2)
Supplement: Supplementary file 16 — Additional file 16. Intervention function and behaviour change techniques identified using the Behaviour Change Taxonomy (Version 1) for each domain included in the behaviour change intervention [file 13012_2021_1122_MOESM16_ESM.docx]

**Additional File16.** Intervention function and behaviour change techniques identified using the Behaviour Change Taxonomy (Version 1)^[[1]](#footnote-1)^ for each domain included in the behaviour change intervention

| **Domains included in the behaviour change intervention** | **TDF Domain (COM-B Domain)** | **Target level:**  **Patients/**  **Practitioners/**  **Services/**  **Community** | **Intervention function** | **Behaviour change techniques** |
| --- | --- | --- | --- | --- |
| 1. How essential is it that patients know about the psychological treatment that he/she could be receiving over the telephone? | Knowledge  (Capabilities - psychological) | Patients | *Education  *Training  *Modelling  *Enablement (services) | 2.2 Feedback on behaviour  4.1 Instruction on how to perform the behaviour  6.1 Demonstration of behaviour  8.1 Behavioural practice rehearsal  8.3 Habit formation |
| 2. How essential is it that patients know telephone appointments are as formal and important as face-to-face? | Knowledge  (Capabilities - psychological) | Patients | *Education  *Training  *Modelling | 2.2 Feedback on behaviour  4.1 Instruction on how to perform the behaviour  6.1 Demonstration of behaviour  8.1 Behavioural practice rehearsal |
| 3. How essential is it that patients know the missed appointment/discharge rules for treatment delivered over the telephone are the same as for face-to-face treatment? | Knowledge  (Capabilities - psychological) | Patients | *Enablement (services) | 4.1. Instruction on how to perform the behaviour |
| 4. How essential is it that patients know treatment over the telephone is being delivered by the same qualified practitioners who deliver face-to-face treatments? | Knowledge  (Capabilities - psychological) | Patients | *Education  *Training  *Enablement (services) | 4.1 Instruction on how to perform a behaviour  5.1 Information about health consequences |
| 5. How essential is it that patients know he/she should answer the session phone calls in a private, quiet and confidential place? | Knowledge  (Capabilities - psychological) | Patients | *Education  *Training  *Modelling  *Enablement (services) | 4.1 Instruction on how to perform a behaviour  5.1 Information about health consequences |
| 7. How essential is it that patients know practitioners might be typing notes into the computer during the telephone session? | Knowledge  (Capabilities - psychological) | Patients | *Education  *Training  *Modelling |  |
| 8. How essential is it for practitioners to have an overall knowledge about the delivery of psychological treatment over the telephone? | Knowledge  (Capabilities - psychological) | Practitioners | *Education  *Training | 1.6 Discrepancy between current behaviour and goal  5.1 Information about health consequences  13.3 Incompatible beliefs |
| 9. How essential is it for practitioners to know about patient experiences of receiving treatment over the telephone? | Knowledge  (Capabilities - psychological) | Practitioners | *Education  *Training  *Enablement | 5.1 Information about health consequences |
| 10. How essential is it for practitioners to know about other practitioner experiences of delivering treatment over the telephone? | Knowledge  (Capabilities - psychological) | Practitioners | *Education  *Training  *Enablement (service) | 5.3 Information about social and environmental consequences  5.6 Information about emotional Consequences  9.1 Credible source |
| 11. How essential is it for practitioners to know about the clinical effectiveness of treatment delivered over the telephone in comparison to face-to-face? | Knowledge  (Capabilities - psychological) | Practitioners | *Education  *Training  *Enablement (service) | 5.1 Information about health consequences |
| 12. How essential is it for practitioners to have 'specific' guidelines to deliver telephone treatment? | Knowledge  (Capabilities - psychological) | Practitioners | *Enablement (services) | 4.1 Instruction on how to perform a behaviour |
| 13. How essential is it to develop/adapt practitioners’ skill-set to deliver treatment over the telephone? | Skills [Communication]  (Capabilities - psychological) | Practitioners | *Education  *Training  *Modelling | 2.2 Feedback on behaviour  4.1 Instruction on how to perform the behaviour  6.1 Demonstration of behaviour  8.1 Behavioural practice rehearsal  8.3 Habit formation |
| 14. How essential is it to develop/adapt practitioners’ verbal communication skills to suit treatment delivered over the telephone (e.g. verbal empathy)? | Skills  [Communication]  (Capabilities - psychological) | Practitioners | *Education  *Training  *Modelling | 2.2 Feedback on behaviour  4.1 Instruction on how to perform the behaviour  6.1 Demonstration of behaviour  8.1 Behavioural practice rehearsal  8.3 Habit formation |
| 15. How essential is it for practitioners to develop skills to adjust their tone of voice over the telephone? | Skills [Communication]  (Capabilities - psychological)) | Practitioners | *Education  *Training  *Modelling | 2.2 Feedback on behaviour  4.1 Instruction on how to perform the behaviour  6.1 Demonstration of behaviour  8.1 Behavioural practice rehearsal  8.3 Habit formation |
| 16. How essential is it for practitioners to develop skills to use and deal with silences when talking over the telephone and to recognise signals to know when to talk and when not to talk? | Skills [Communication]  (Capabilities - psychological) | Practitioners | *Education  *Training  *Modelling | 2.2 Feedback on behaviour  4.1 Instruction on how to perform the behaviour  6.1 Demonstration of behaviour  8.1 Behavioural practice rehearsal  8.3 Habit formation |
| 17. How essential is it for practitioners to develop skills to create a feeling/sense of a ‘therapeutic space’ and a ‘safe space to talk’ when delivering treatment over the telephone? | Skills  [Opening + treatment]  (Capabilities - psychological) | Practitioners | *Education  *Training  *Modelling  *Enablement (services) | 2.2. Feedback on behaviour  4.1 Instruction on how to perform a behaviour  6.1 Demonstration of behaviour  8.1 Behavioural practice/rehearsal  8.3 Habit formation |
| 18. How essential is it for practitioners to develop skills to build up a good relationship with patients over the telephone? | Skills  [Opening + treatment]  (Capabilities - psychological) | Practitioners | *Education  *Training  *Modelling  *Enablement (services) | 2.2. Feedback on behaviour  4.1 Instruction on how to perform a behaviour  6.1 demonstration of behaviour  8.1 Behavioural practice/rehearsal  8.3 Habit formation |
| 19. How essential is it for practitioners to convey active listening when they are delivering treatment over the telephone? | Skills [Communication]  (Capabilities - psychological) | Practitioners | *Education  *Training  *Modelling  *Enablement (services) | 2.2. Feedback on behaviour  4.1 Instruction on how to perform a behaviour  6.1 Demonstration of behaviour  8.1 Behavioural practice/rehearsal  8.3 Habit formation |
| 20. How essential is it for practitioners to develop skills to personalise and adapt the treatment to patient's individual need when delivering treatment over the telephone? | Skills  [Specific]  (Capabilities - psychological) | Practitioners | *Education  *Training  *Modelling  *Enablement (services) | 2.2. Feedback on behaviour  4.1 Instruction on how to perform a behaviour  6.1 demonstration of behaviour  8.1 Behavioural practice/rehearsal  8.3 Habit formation |
| 21. How essential is it for practitioners to develop skills to help the patient understand the formulation of their current difficulties and set up homework without visual aids (e.g. diagrams)? | Skills  [Specific]  (Capabilities - psychological) | Practitioners | *Education  *Training  *Modelling  *Enablement (services) | 2.2. Feedback on behaviour  4.1 Instruction on how to perform a behaviour  6.1 demonstration of behaviour  8.1 Behavioural practice/rehearsal  8.3 Habit formation |
| 22. How essential is it for practitioners to develop skills to work collaboratively with patients over the telephone without visual aids (e.g. diagrams)? | Skills  [Specific]  (Capabilities - psychological) | Practitioners | *Education  *Training  *Modelling  *Enablement (services) | 2.2. Feedback on behaviour  4.1 Instruction on how to perform a behaviour  6.1 demonstration of behaviour  8.1 Behavioural practice/rehearsal  8.3 Habit formation |
| 23. How essential is it for practitioners to develop skills to use the symptom questionnaires in an integrative way when they are working over the telephone (e.g. using patient questionnaire answers to decide the focus of the session)? | Skills  [Specific]  (Capabilities - psychological) | Practitioners | *Education  *Training  *Modelling | 2.2 Feedback on behaviour  4.1 Instruction on how to perform a behaviour  6.1 demonstration of behaviour  8.1 behavioural practice rehearsal  8.3 Habit formation  8.4 Habit reversal |
| 24. How essential is it for practitioners to develop skills to assess patient suitability for telephone treatment (e.g. learning difficulties, hearing problems)? | Skills  [Specific]  (Capabilities - psychological) | Practitioners | *Education  *Training  *Modelling  *Enablement (services) | 2.2 Feedback on behaviour  4.1 Instruction on how to perform the behaviour  6.1 demonstration of behaviour  8.1 behavioural practice rehearsal  8.3 Habit formation |
| 25. How essential is it for practitioners to develop skills to explore, address and manage patient expectations regarding treatment delivered over the telephone? | Skills  [Opening + treatment]  (Capabilities - psychological) | Practitioners | *Education  *Training  *Modelling  *Enablement (services) | 2.2. Feedback on behaviour  4.1 Instruction on how to perform a behaviour  6.1 demonstration of behaviour  8.1 Behavioural practice/rehearsal  8.3 Habit formation |
| 26. How essential is it for practitioners to develop skills to explore patient feelings and thoughts about working over the telephone? | Skills  [Opening + treatment]  (Capabilities - psychological) | Practitioners | *Education  *Training  *Modelling | 2.2 Feedback on behaviour  4.1 Instruction on how to perform a behaviour  6.1 demonstration of behaviour  8.1 behavioural practice rehearsal  8.3 Habit formation  8.4 Habit reversal |
| 27. How essential is it for practitioners to develop skills to help patients to reflect/express their experiences of working over the telephone? | Skills  [Opening + treatment]  (Capabilities - psychological) | Practitioners | *Education  *Training  *Modelling | 2.2 Feedback on behaviour  4.1 Instruction on how to perform a behaviour  6.1 Demonstration of the behaviour  8.1 Behavioural practice/ rehearsal  8.3 Habit formation |
| 28. How essential is it for practitioners to develop skills to contain more talkative patients and manage less talkative patients when the intervention is delivered over the telephone? | Skills  [Communication]  (Capabilities - psychological) | Practitioners | *Education  *Training  *Modelling | 2.2. Feedback on behaviour  4.1 Instruction on how to perform a behaviour  6.1 Demonstration of behaviour  8.7 Graded tasks |
| 29. How essential is it for practitioners to develop skills to gauge patient understanding and monitor patient progress when the intervention is delivered over the telephone? | Skills  [Opening + treatment]  (Capabilities - psychological) | Practitioners | *Education  *Training  *Modelling  *Enablement (services) | 2.2. Feedback on behaviour  4.1 Instruction on how to perform a behaviour  6.1 demonstration of behaviour  8.1 Behavioural practice/rehearsal  8.3 Habit formation |
| 30. How essential is it for practitioners to develop skills to increase patient commitment and motivation to change over the telephone WITHIN the session? | Skills  [Opening + treatment]  (Capabilities - psychological) | Practitioners | *Education  *Training  *Modelling | 2.2 Feedback on behaviour  4.1 Instruction on how to perform a behaviour  6.1 demonstration of behaviour  8.1 Behavioural practice/rehearsal  8.3 Habit formation |
| 31. How essential is it for practitioners to develop skills to increase patient commitment and motivation to change over the telephone BETWEEN sessions (enhance links between sessions)? | Skills  [Opening + treatment]  (Capabilities - psychological) | Practitioners | *Education  *Training  *Modelling | 2.2 Feedback on behaviour  4.1 Instruction on how to perform a behaviour  6.1 demonstration of behaviour  8.1 Behavioural practice/rehearsal  8.3 Habit formation |
| 32. How essential is it for practitioners to develop skills to manage homework non-compliance over the telephone? | Skills  [Specific]  (Capabilities - psychological) | Practitioners | *Education  *Training  *Modelling  *Enablement (services) | 2.2. Feedback on behaviour  4.1 Instruction on how to perform a behaviour  6.1 demonstration of behaviour  8.1 Behavioural practice/rehearsal  8.3 Habit formation |
| 33. How essential is it for practitioners to develop skills to deal with noises and other potential issues related to the patient's surroundings when answering the session phone call (e.g. supermarket, park, baby crying)? | Skills [Communication]  (Capabilities - psychological) | Practitioners | *Education  *Training  *Modelling | 2.2 Feedback on behaviour  4.1 Instruction on how to perform the behaviour  6.1 demonstration of behaviour  8.1 Behavioural practice rehearsal  8.3 Habit formation |
| 34. How essential is it for practitioners to develop skills to cope with telephone work demands (e.g. time constraints, number of clinical cases)? | Skills  (Capabilities - psychological) | Services | *Enablement (services) | 4.1 Instruction on how to perform a |
| 35. How essential is it for practitioners to reflect on their attitudes and beliefs related to delivering treatment over the telephone? | Belief about consequences  [Reflection]  (Motivation – reflective) | Practitioners | *Education  *Training  *Enablement (services) | 2.3 Self-monitoring of behaviour  2.4 Self-monitoring of outcome(s)  of behaviour  4.1 Instruction on how to perform a behaviour  6.2 Social comparison  7.1 Prompts/cues  12.5 Adding objects to the environment |
| 36. How essential is it for practitioners to reflect on the benefits of telephone treatment (including for the patients, practitioners, service)? | Reinforcement  [Reflection]  (Motivation – automatic) | Practitioners | *Education  *Training  *Enablement (services) | 2.3 Self-monitoring of behaviour  2.4 Self-monitoring of outcome(s) of behaviour  4.1 Instruction on how to perform a behaviour  6.2 Social comparison  7.1 Prompts/cues (physical sticker on the PC)  12.5 Adding objects to the environment |
| 37. How essential is it for practitioners to reflect on the practical reasons services deliver assessments and treatments over the telephone compared to the health care guidelines/evidence-base available for its use? | Belief about consequences  [Reflection]  (Motivation – reflective) | Practitioners | *Enablement (services) | 4.1 Instruction on how to perform the behaviour |
| 38. How essential is it for practitioners to reflect on patient attitudes, views and preconceptions of telephone treatment and how to improve patient commitment to working together over the telephone? | Belief about consequences  [Reflection]  (Motivation – reflective) | Practitioners | *Education  *Training  *Enablement (services) | 2.3 Self-monitoring of behaviour  2.4 Self-monitoring of outcome(s) of behaviour  4.1 Instruction on how to perform a behaviour  6.2 Social comparison  7.1 Prompts/cues (physical sticker on the PC)  12.5 Adding objects to the environment |
| 39. How essential is it for practitioners to reflect on their attitude, views and preconceptions of telephone treatment and how to improve their commitment towards this mode of working? | Belief about consequences  [Reflection]  (Motivation – reflective) | Practitioners | *Education  *Persuasion  *Enablement (services) | 2.3 Self-monitoring of behaviour (performance)  2.4 Self-monitoring of outcomes of behaviour (in terms of patient outcomes)  4.1 Instruction on how to perform a behaviour  6.2 Social comparison (add in the other items with attitudes/beliefs)  7.1 Prompts/cues  12.5 Adding objects to the environment |
| 40. How essential is it for practitioners to reflect on what other mental health professionals think and feel about the delivery of treatment over the telephone and how to improve their views towards it? | Belief about consequences  [Reflection]  (Motivation – reflective) | Practitioners | *Enablement (services) | 4.1 Instruction on how to perform the behaviour |
| 41. How essential is it for practitioners to challenge their own, their patients’ or other people’s beliefs about treatment delivered over the telephone (e.g. ‘Treatment delivered over the telephone is a lower and cheaper version of therapy’)? | Belief about consequences  [Reflection]  (Motivation – reflective) | Practitioners | *Education  *Training  *Enablement (services) | 2.3 Self-monitoring of behaviour  2.4 Self-monitoring of outcome(s) of behaviour  4.1 Instruction on how to perform a behaviour  6.2 Social comparison  7.1 Prompts/cues  12.5 Adding objects to the environment |
| 42. How essential is it for practitioners to overcome personal dislike of treatment delivered over the telephone through training? | Belief about consequences  [Reflection]  (Motivation – reflective) | Practitioners | *Education  *Training | 1.6 Discrepancy between current behaviour and goal  5.1 Information about health consequences  13.3 Incompatible beliefs |
| 43. How essential is it for practitioners to overcome any personal dislike of treatment delivered over the telephone through practice? | Belief about consequences  [Reflection]  (Motivation – reflective) | Practitioners | *Education  *Training | 1.6 Discrepancy between current behaviour and goal  5.1 Information about health consequences  13.3 Incompatible beliefs |
| 44. How essential is it for practitioners to discuss audios of telephone treatment sessions during university training to assess and improve your performance? | Beliefs about capabilities  [Reflection + Skills]  (Motivation – reflective + Capabilities - psychological) | Universities | *Enablement (university training courses) | 2.2 Feedback on behaviour  4.1 Instruction on how to perform a behaviour |
| 45. How essential is it for practitioners to be assessed on telephone specific abilities at university training before they become qualified? | Beliefs about capabilities  (Motivation – reflective + Capabilities - psychological) | Universities | *Enablement (university training courses) | 4.1 Instruction on how to perform a behaviour |
| 46. How essential is it for practitioners to record telephone treatment sessions with patient agreement to reflect on the session to improve performance? | Beliefs about capabilities  (Motivation – reflective + Capabilities - psychological) | Services | *Enablement (service) | 4.1 Instruction on how to perform a behaviour  *If they are able to record, then*  2.3 Self-monitoring of behaviour |
| 47. How essential is it for practitioners to discuss their professional role expectations with service managers and colleagues, and whether these fit with the reality of their day-to-day work? | Professional role  [Reflection]  (Motivation – reflective) | Services  Universities | *Enablement (service and univ) | 4.1 Instruction on how to perform the behaviour |
| 48. How essential is it for practitioners to reflect on their role as a coach or as a therapist? | Professional role  [Reflection]  (Motivation – reflective) | Practitioners | *Education  *Training | 1.6 Discrepancy between current behaviour and goal  5.1 Information about health consequences  13.3 Incompatible beliefs |
| 51. How essential is it for practitioners to reflect on their feelings regarding delivering treatment over the telephone? | Professional role/Emotion  [Reflection]  (Motivation – reflective/automatic) | Practitioners | *Education  *Training  *Enablement (services) | 2.3 Self-monitoring of behaviour  2.4 Self-monitoring of outcome(s) of behaviour  4.1 Instruction on how to perform a behaviour  6.2 Social comparison  7.1 Prompts/cues (physical sticker on the PC)  12.5 Adding objects to the environment |
| 53. How essential is it for practitioners to discuss with service managers and colleagues about possibly feeling undervalued? | Professional role/Emotion  [Reflection]  (Motivation – reflective/automatic) | Services | *Enablement (services) | 4.1 Instruction on how to perform the behaviour |
| 54. How essential is it for practitioners to be motivated to deliver treatment over the telephone? | Reinforcement  (Motivation –automatic) | Services | *Persuasion  *Enablement (service) |  |
| 55. How essential is it for practitioners to know that patients had a positive experience of telephone treatment to increase their motivation to continue delivering treatment over the telephone? | Reinforcement  (Motivation –automatic) | Services | *Training  *Enablement (services) | 2.3 Self-monitoring behaviour |
| 56. How essential is it for practitioners to feel motivated to deliver treatment over the phone, by having the ability to offer choices to patients about how they want to receive treatment (e.g., telephone, face-to-face, group)? | Reinforcement  (Motivation –automatic) | Services | *Enablement (services) | 5.3 Information about social and environmental consequences  14.10 Remove punishment |
| 57. How essential is it that NHS services provide practitioners with 'specific' training on treatment delivered over the telephone before they start using this mode of delivery? | Knowledge  [from services to practitioners]  (Capabilities – psychological) | Services | *Enablement (services) | 4.1 Instruction on how to perform the behaviour |
| 58. How essential is it that NHS services provide practitioners with information related to sharing materials with patients when the intervention is delivered over the telephone (post, email, before/after the session, workbook/worksheet)? | Knowledge  [from services to practitioners]  (Capabilities – psychological) | Services | *Enablement (services) | 4.1 Instruction on how to perform the behaviour |
| 59. How essential is it that NHS services provide practitioners with information about how to proceed over the telephone if they are concerned about patient welfare/safety if your patient is at risk (procedures in place if practitioners are working at the service and if they are working from home)? | Knowledge  [from services to practitioners]  (Capabilities – psychological) | Services | *Education  *Training  *Modelling  *Enablement (services) | 2.2 Feedback on behaviour  4.1 Instruction on how to perform the behaviour  6.1 demonstration of behaviour  8.1 behavioural practice rehearsal  8.3 Habit formation |
| 60. How essential is it that NHS services provide practitioners with information about discharge procedures for treatments delivered over the telephone? | Knowledge  [from services to practitioners]  (Capabilities – psychological) | Services | *Enablement (services) |  |
| 61. How essential is it that NHS services provide practitioners with information about how to proceed if the patient does not answer the phone call or in case phone contact/communication is lost in the middle of a session (e.g. number of times to call back, leaving a voice mail)? | Knowledge  [from services to practitioners]  (Capabilities – psychological) | Services | *Enablement (services)  *Education  *Training | 4.1. Instruction on how to perform the behaviour |
| 62. How essential is it that NHS services provide practitioners with information about how to proceed if the patient answers the call from a supermarket or a park, places that are not confidential and private? | Knowledge  [from services to practitioners]  (Capabilities – psychological) | Services | *Enablement (services) | 4.1 Instruction on how to perform the behaviour |
| 63. How essential is it that NHS services provide practitioners with information about how to proceed with homework non-compliance? | Knowledge  [from services to practitioners]  (Capabilities – psychological) | Services | *Enablement (services) | 4.1 Instruction on how to perform the behaviour |
| 64. How essential is the working environment in which practitioners deliver treatment over the telephone? | Environmental context & Resources  (Opportunity – physical) | Services | *Enablement (services) | 12.1 Restructuring the physical environment |
| 65. How essential is it that the working environment facilitates active listening (e.g. remove distractions)? | Environmental context & Resources  (Opportunity – physical) | Services | *Enablement (services) | 12.1 Restructuring the physical environment |
| 71. How essential is it that NHS services count with the resources/equipment that are needed to deliver treatment over the telephone? | Environmental context & Resources  (Opportunity – physical) | Services | *Enablement (services) | 4.1 Instruction on how to perform the behaviour |
| 72. How essential is it that NHS services have different options available to share materials with patients to meet their needs (e.g. email, post, on-line)? | Environmental context & Resources  (Opportunity – physical) | Services | *Enablement (services) | 4.1 Instruction on how to perform the behaviour |
| 73. How essential is it that a number of headsets and good quality headsets are available within the NHS services to deliver treatment over the telephone? | Environmental context & Resources  (Opportunity – physical) | Services | *Enablement (services) | 12.1 Restructuring the physical environment |
| 74. How essential is it that NHS services offer options to patients so they can decide on how they would like to receive psychological treatment (e.g. face-to-face, telephone, group, online)? | Environmental context & Resources  (Opportunity – physical) | Services | *Enablement (services) | 12.1 Restructuring the physical environment |
| 75. How essential is it that NHS services provide flexibility to offer patients an assessment and/or the first treatment session face-to-face? | Environmental context & Resources  (Opportunity – physical) | Services | *Enablement (services) | 12.1 Restructuring the physical environment |
| 76. How essential is it that NHS services are able to identify the mode of treatment delivery (e.g., face-to-face, telephone) within the electronic databases? | Environmental context & Resources  (Opportunity – physical) | Services | *Enablement (services) | 12.1 Restructuring the physical environment |
| 79. How essential is it that NHS services increase and acknowledge the time it takes practitioners to prepare for sessions being delivered over the telephone? | Environmental context & Resources  (Opportunity – physical) | Services | *Enablement (services) | 12.1 Restructuring the physical environment |
| 80. How essential is it that NHS service provide flexibility to deliver treatment using different modalities and not mainly telephone, i.e. face-to-face and telephone? | Environmental context & Resources  (Opportunity – physical) | Services | *Enablement (services) | 12.1 Restructuring the physical environment |
| 81. How essential is it that NHS services provide support to deliver treatment over the telephone? | Social Influences  (Opportunity – social) | Services | *Enablement (service) |  |
| 82. How essential is it that NHS services are able to have arrangements in place to allow practitioners joining the service to observe/shadow an experienced colleague working over the telephone before delivering treatment over the telephone? | Social Influences  (Opportunity – social) | Services | *Enablement (service) | 3.1 Social support  3.2 Social support (practical)  3.3 Social support (emotional)  6.3 Information about other's approval  10.4 Social reward |
| 83. How essential is it that NHS services provide INITIAL close supervision to assess your telephone skills and performance on the delivery of treatment over the telephone? | Social Influences  (Opportunity – social) | Services | *Enablement (services) | 4.1 Instruction on how to perform the behaviour |
| 84. How essential is it that NHS services provide regular supervision to support practitioners on the development of telephone skills and increasing their confidence? | Social Influences  (Opportunity – social) | Services | *Enablement (services) | 4.1 Instruction on how to perform the behaviour |
| 87. How essential is it that clinical managers have experience delivering treatment over the telephone? | Social Influences  (Opportunity – social) | Services | *Enablement (services) | 9.1 Credible source |
| 88. How essential is it that clinical managers have a positive view/attitude towards treatment delivered over the telephone? | Social Influences  (Opportunity – social) | Services | *Enablement (service) | 5.3 Information about social and environmental consequences  5.6 Information about emotional consequences  6.3 Information about others approval |
| 89. How essential is it for practitioners to receive support to reflect on reasons that might be affecting/interfering with patient recovery over the telephone (e.g. not blame the telephone without having a strong reason/logic for it)? | Social Influences (Opportunity – social) | Services | *Enablement (services) | 4.1 Instruction on how to perform the behaviour |
| 90. How essential is it for practitioners to have opportunities with NHS services for professional development (ongoing training, booster skills sessions)? | Social Influences  (Opportunity – social) | Services | *Enablement (services) | 4.1 Instruction on how to perform the behaviour |
| 91. How essential is it for practitioners that NHS services promote working together as a team and facilitate peer support and advice about treatment delivered over the telephone? | Social Influences  (Opportunity – social) | Services | *Enablement (services) | 12.1 Restructuring the physical environment |
| 92. How essential is it that GPs are knowledgeable about the IAPT psychological treatments they refer patients to? | Social Influences  (Opportunity – social) | Community | *Enablement | 4.1 Instruction on how to perform the behaviour |
| 93. How essential is it that the public is aware of the variety of different psychological treatments (e.g. not just counselling) and different methods/modes of delivery (e.g. not just face-to-face)? | Social Influences  (Opportunity – social) | Community | *Enablement | 4.1 Instruction on how to perform the behaviour |

1. Michie S, Richardson M, Johnston M, Abraham C, Francis J, Hardeman W, Eccles MP, Cane J, Wood CE. The behavior change technique taxonomy (v1) of 93 hierarchically clustered techniques: building an international consensus for the reporting of behavior change interventions. Ann Behav Med. 2013; 46(1):81-95. [↑](#footnote-ref-1)
