# Supplementary material for: Enhancing the Behaviour Change Wheel with synthesis, stakeholder involvement and decision-making: a case example using the ‘Enhancing the Quality of Psychological Interventions Delivered by Telephone’ (EQUITy) research programme
Source: Implement Sci. 2021 May 14;16:53. doi: 10.1186/s13012-021-01122-2 (PMC8120925; doi:10.1186/s13012-021-01122-2)
Supplement: Supplementary file 17 — Additional file 17. The Template for Intervention Description and Replication (TIDieR) Checklist [file 13012_2021_1122_MOESM17_ESM.docx]

**Additional File 17.** The Template for Intervention Description and Replication (TIDieR) Checklist^[[1]](#footnote-1)^

**
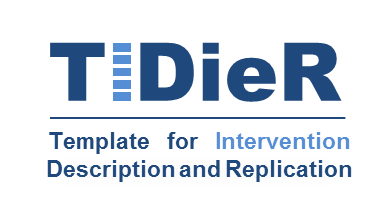
The TIDieR (Template for Intervention Description and Replication) Checklist*:**

Information to include when describing an intervention and the location of the information

| **Item number** | **Item** | **Where located **** | | |
| --- | --- | --- | --- | --- |
|  |  | Primary paper  (page or appendix  number) | Other ^†^ (details) |  |
|  | **BRIEF NAME** |  |  |  |
| **1.** | Provide the name or a phrase that describes the intervention. | Enhancing the quality of psychological interventions delivered by telephone  (page 6, lines 128-129) | ______________ |  |
|  | **WHY** |  |  |  |
| **2.** | Describe any rationale, theory, or goal of the elements essential to the intervention. | Elements of the intervention and goals: (page 8 lines 185-194, page 12 lines 291-293, pages 16-17 lines 405-413, Figure 3, Table 7, Additional File 8)  1) *Recommendations for mental health services.* The aim is to help services improve the quality and ease of delivering telephone-based psychological interventions, and facilitate the implementation of suggested recommendations.  2) *Telephone training for practitioners*. The aim is to enhance practitioners’ telephone specific skills.  3) *Resources for patients and community*. The aim is to increase awareness in psychological interventions and its different modes of delivery (e.g. telephone). | _____________ |  |
|  | **WHAT** | Elements of the intervention and goals: (page 12 lines 291-293, Figure 3, Table 7, Additional File 8) | **Note:** As the behaviour change intervention has not yet been tested (feasibility or large scale trial) materials are not available at this stage but will be made available at a later stage when testing is complete. |  |
| **3.** | Materials: Describe any physical or informational materials used in the intervention, including those provided to participants or used in intervention delivery or in training of intervention providers. Provide information on where the materials can be accessed (e.g. online appendix, URL). | 1) *Recommendations for services* *materials:* a booklet highlighting 5 key areas to improve telephone delivery (i.e. promoting telephone work, incorporating key elements of telephone work, working environment and resources, boosting practitioner telephone skills, and reflection) and short video-clips to facilitate implementation of the intervention  2) *Training for practitioners: a* two-full day training  3) *Resources for patients materials*: a leaflet, a poster, and an appointment card |  |  |
| **4.** | Procedures: Describe each of the procedures, activities, and/or processes used in the intervention, including any enabling or support activities. | 1) *Recommendations for services:* short video clips to facilitate implementation of the suggested changes to improve telephone delivery  2) *Training for practitioners:* session slides, practitioner manual, classroom session, group discussion, skills practice using clinical vignettes.  *3) Resources for patients:* leaflet and poster to be used in mental health services, GP surgeries, community. |  |  |
|  | **WHO PROVIDED** |  |  |  |
| **5.** | For each category of intervention provider (e.g. psychologist, nursing assistant), describe their expertise, background and any specific training given. | N/A | **Note:** As the behaviour change intervention has not yet been tested (feasibility or large scale trial) items 5 to 12 are reported as N/A. These details will be provided at a later stage when testing is complete. |  |
|  | **HOW** |  |  |  |
| **6.** | Describe the modes of delivery (e.g. face-to-face or by some other mechanism, such as internet or telephone) of the intervention and whether it was provided individually or in a group. | N/A | _____________ |  |
|  | **WHERE** |  |  |  |
| **7.** | Describe the type(s) of location(s) where the intervention occurred, including any necessary infrastructure or relevant features. | N/A | _____________ |  |
|  | **WHEN and HOW MUCH** |  |  |  |
| **8.** | Describe the number of times the intervention was delivered and over what period of time including the number of sessions, their schedule, and their duration, intensity or dose. | N/A | _____________ |  |
|  | **TAILORING** |  |  |  |
| **9.** | If the intervention was planned to be personalised, titrated or adapted, then describe what, why, when, and how. | N/A | _____________ |  |
|  | **MODIFICATIONS** |  |  |  |
| **10.^ǂ^** | If the intervention was modified during the course of the study, describe the changes (what, why, when, and how). | N/A | _____________ |  |
|  | **HOW WELL** |  |  |  |
| **11.** | Planned: If intervention adherence or fidelity was assessed, describe how and by whom, and if any strategies were used to maintain or improve fidelity, describe them. | N/A | _____________ |  |
| **12.^ǂ^** | Actual: If intervention adherence or fidelity was assessed, describe the extent to which the intervention was delivered as planned. | N/A | _____________ |  |

** **Authors** - use N/A if an item is not applicable for the intervention being described. **Reviewers** – use ‘?’ if information about the element is not reported/not sufficiently reported.

† If the information is not provided in the primary paper, give details of where this information is available. This may include locations such as a published protocol or other published papers (provide citation details) or a website (provide the URL).

ǂ If completing the TIDieR checklist for a protocol, these items are not relevant to the protocol and cannot be described until the study is complete.

* We strongly recommend using this checklist in conjunction with the TIDieR guide (see *BMJ* 2014;348:g1687) which contains an explanation and elaboration for each item.

* The focus of TIDieR is on reporting details of the intervention elements (and where relevant, comparison elements) of a study. Other elements and methodological features of studies are covered by other reporting statements and checklists and have not been duplicated as part of the TIDieR checklist. When a **randomised trial** is being reported, the TIDieR checklist should be used in conjunction with the CONSORT statement (see [www.consort-statement.org](http://www.consort-statement.org)) as an extension of **Item 5 of the CONSORT 2010 Statement.** When a **clinical trial** **protocol** is being reported, the TIDieR checklist should be used in conjunction with the SPIRIT statement as an extension of **Item 11 of the SPIRIT 2013 Statement** (see [www.spirit-statement.org](http://www.spirit-statement.org)). For alternate study designs, TIDieR can be used in conjunction with the appropriate checklist for that study design (see [www.equator-network.org](http://www.equator-network.org)).

1. Hoffmann TC, Glasziou PP, Boutron I, Milne R, Perera R, Moher D, et al. Better reporting of interventions: Template for intervention description and replication (TIDieR) checklist and guide. BMJ. 2014;348. g1687 [↑](#footnote-ref-1)
